# Drivers of aboveground wood production in a lowland tropical forest of West Africa: teasing apart the roles of tree density, tree diversity, soil phosphorus, and historical logging

**DOI:** 10.1002/ece3.2175

**Published:** 2016-05-18

**Authors:** Tommaso Jucker, Aida Cuni Sanchez, Jeremy A. Lindsell, Harriet D. Allen, Gabriel S. Amable, David A. Coomes

**Affiliations:** ^1^ Forest Ecology and Conservation GroupDepartment of Plant SciencesUniversity of CambridgeDowning StreetCambridgeCB2 3EAUK; ^2^ RSPB Centre for Conservation ScienceThe LodgeSandyBedfordshireSG19 2DLUK; ^3^ Department of BiologyCenter for Macroecology, Evolution and ClimateUniversity of CopenhagenUniversitetsparken 15DK‐2100CopenhagenDenmark; ^4^ A Rocha International89 Worship StreetLondonEC2A 2BFUK; ^5^ Department of GeographyUniversity of CambridgeDowning PlaceCambridgeCB2 3ENUK

**Keywords:** Determinants of plant community diversity and structure, effective number of species, forest productivity, packing density, selective logging, Sierra Leone, soil nutrients, structural equation modeling

## Abstract

Tropical forests currently play a key role in regulating the terrestrial carbon cycle and abating climate change by storing carbon in wood. However, there remains considerable uncertainty as to whether tropical forests will continue to act as carbon sinks in the face of increased pressure from expanding human activities. Consequently, understanding what drives productivity in tropical forests is critical. We used permanent forest plot data from the Gola Rainforest National Park (Sierra Leone) – one of the largest tracts of intact tropical moist forest in West Africa – to explore how (1) stand basal area and tree diversity, (2) past disturbance associated with past logging, and (3) underlying soil nutrient gradients interact to determine rates of aboveground wood production (AWP). We started by statistically modeling the diameter growth of individual trees and used these models to estimate AWP for 142 permanent forest plots. We then used structural equation modeling to explore the direct and indirect pathways which shape rates of AWP. Across the plot network, stand basal area emerged as the strongest determinant of AWP, with densely packed stands exhibiting the fastest rates of AWP. In addition to stand packing density, both tree diversity and soil phosphorus content were also positively related to productivity. By contrast, historical logging activities negatively impacted AWP through the removal of large trees, which contributed disproportionately to productivity. Understanding what determines variation in wood production across tropical forest landscapes requires accounting for multiple interacting drivers – with stand structure, tree diversity, and soil nutrients all playing a key role. Importantly, our results also indicate that logging activities can have a long‐lasting impact on a forest's ability to sequester and store carbon, emphasizing the importance of safeguarding old‐growth tropical forests.

## Introduction

By sequestering CO_2_ from the atmosphere and storing it in wood, tropical forests currently act as a net carbon sink and play a critical role in abating climate change (Pan et al. 2011). However, whether this carbon sink will persist into the future remains unclear (Clark et al. 2003; Baker et al. 2004; Feeley et al. 2007; Lewis et al. 2009; Dong et al. 2012; Brienen et al. 2015), especially as tropical forests continue to be threatened by human activities (Laurance 1999; Chazdon 2003; Asner et al. 2009). Part of this uncertainty stems from the fact that while multiple drivers are known to influence rates of aboveground wood production (AWP) in tropical forests (e.g., climate, soils, forest structure, functional traits, human disturbance; Malhi et al. 2004; Asner et al. 2009; Banin et al. 2014; Lasky et al. 2014), few studies have considered how these drivers act together to shape AWP. Consequently, we continue to lack a clear understanding of the multiple interacting factors which together control AWP, especially in the context of the African tropics which remain relatively understudied (Lewis et al. 2009; Cleveland et al. 2011; Banin et al. 2014).

A number of biotic and abiotic factors have been shown to be important in driving AWP in forests. For instance, the number and mean size of trees in a given patch of forest – which together determine the basal area of the stand – are strongly tied to aboveground biomass and forest structure, both of which are key drivers of AWP (Keeling and Phillips 2007; Slik et al. 2010; Hardiman et al. 2011; Coomes et al. 2014; Jenkins 2015). Tree diversity has also been shown to be an important driver of forest AWP, as complementary ecological strategies among co‐occurring species enable trees to use resources more efficiently and pack more densely in space (Chisholm et al. 2013; Vilà et al. 2013; Jucker et al. 2014, 2015; Lasky et al. 2014; Pretzsch 2014). In addition to forest structure and composition, carbon sequestration in forests is also controlled by the abiotic environment (e.g., Boisvenue and Running 2006). In tropical rain forests, soil nutrients (phosphorus in particular) have been shown to play a central role in shaping both large and fine‐scale variation in forest AWP (Banin et al. 2014), in some cases even more so than climate (Malhi et al. 2004; Cleveland et al. 2011). Lastly, forest disturbance associated with human activities such as logging, mining, and land conversion is cause for concern across the tropics (Asner et al. 2009). Logging, for example, can impact AWP in a number of ways, including damaging live trees and altering the structure of the canopy (Okuda et al. 2003; Asner et al. 2004; Blanc et al. 2009; West et al. 2014), through soil impoverishment as a result of erosion and nutrient leaching (Chazdon 2003), and by facilitating the establishment of lianas (Schnitzer and Bongers 2011; Durán et al. 2013). One process in particular – the removal of large diameter trees (Okuda et al. 2003; Bonnell et al. 2011; Osazuwa‐Peters et al. 2015) – can have a sizable and long‐lasting impact on AWP, as large trees contribute disproportionately to productivity (Slik et al. 2013; Michaletz et al. 2014; Stephenson et al. 2014) and it can take decades for surviving trees to take their place in the canopy (Martin et al. 2013; Kent et al. 2015; Osazuwa‐Peters et al. 2015).

Here, we used repeat census data from permanent forest plots distributed across Gola Rainforest National Park in Sierra Leone – one of the largest tracts of intact tropical moist forest in West Africa – to explore how the combined effects of stand basal area, tree diversity, soil phosphorus, and past logging shape current patterns of AWP. Using structural equation modeling, we tested the following hypotheses regarding the relative contribution of each of the above drivers to AWP rates: (1) Forest productivity is intrinsically tied to the frequency and mean size of stems, resulting in a strongly positive relationship between basal area and AWP; (2) tree diversity generally promotes AWP; (3) soil phosphorus limits rates of AWP; and (4) selective logging has a long‐lasting impact on AWP through the removal of large trees which disproportionately influence productivity.

## Materials and Methods

### Study site

**Figure 1:**
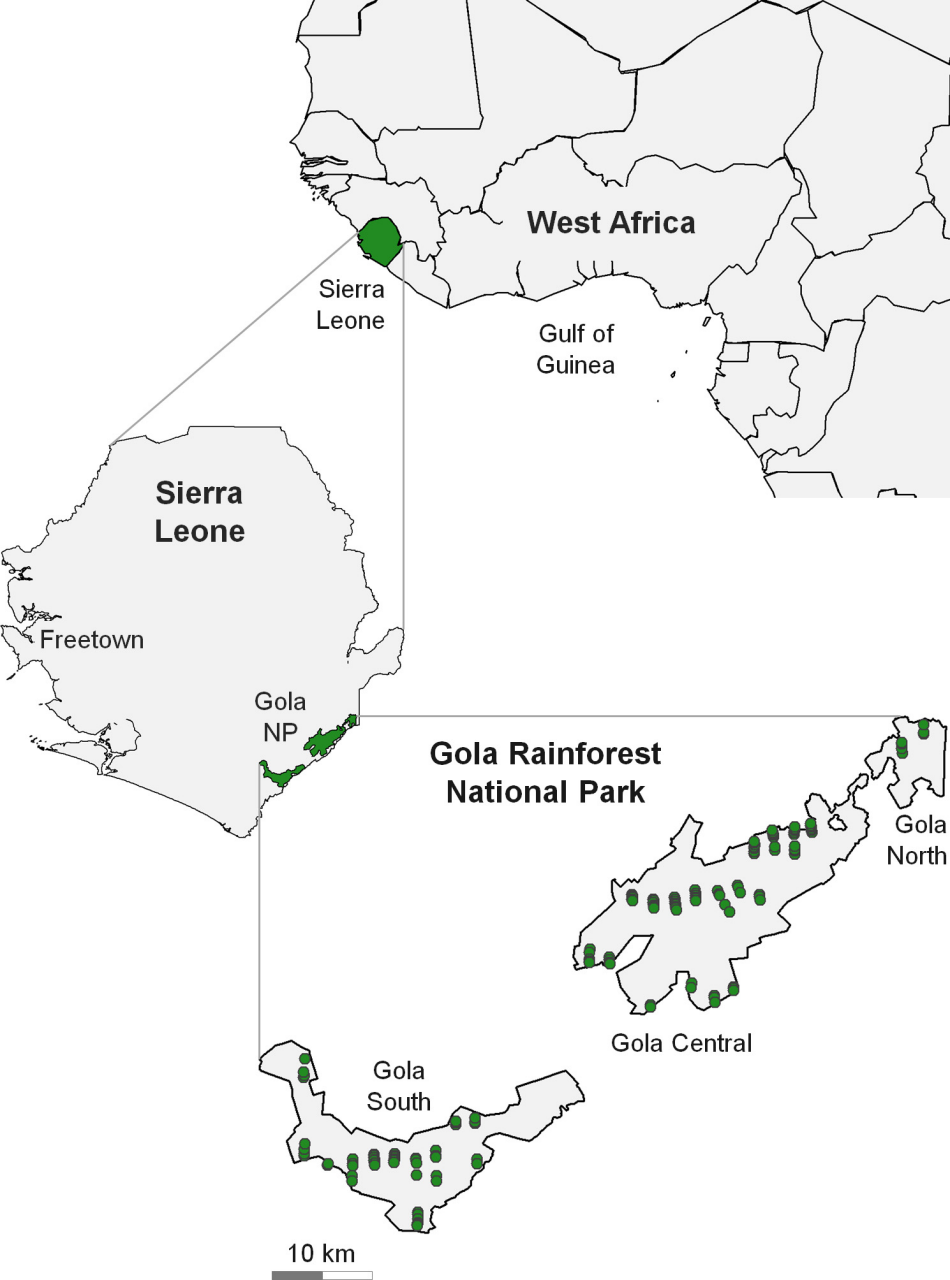
Location of Sierra Leone and the Gola Rainforest National Park. The inset map of Gola shows the location of the 142 permanent forest plots recensused for this study (green circles).

The Gola Rainforest National Park (hereafter “Gola”) lies along the border with Liberia between 7°18′ and 7°51′N and 10°37′ and 11°21′W (Fig. 1). It is the largest remaining area of intact lowland moist evergreen forest in Sierra Leone and is at the western extremity of the Upper Guinea forest block. Annual rainfall is 2500–3000 mm and is mostly concentrated in a single wet season between May and October. The woody vegetation is dominated by Fabaceae (both Caesalpinioideae and Mimosoideae subfamilies), Euphorbiaceae, and Sterculiaceae (Klop et al. 2008). Gola was divided into three forest blocks during the 1930s (see inset in Fig. 1), when commercial logging activities first began in the park (Lindsell and Klop 2013). Gola South (ca. 272 km^2^) is low‐lying and swampy in places (mean elevation 147 m). Gola Central (ca. 417 km^2^) and Gola North (ca. 61 km^2^) are more rugged and at a higher elevation than the surrounding landscape (mean elevation 303 m). Commercial logging activities reached a peak during the 1960s and 1980s, but since the 1990s the park has been the focus of an ongoing conservation project which in 2011 culminated with Gola being declared a national park. Currently the park is managed through a collaborative project between the Government of Sierra Leone, the Conservation Society of Sierra Leone, and the UK's Royal Society for the Protection of Birds (RSPB).

### Permanent plot network

#### Tree inventory data

Between February 2012 and July 2013, we resurveyed 142 permanent forest plots within Gola (Fig. 1). Plots were circular with a radius of 19.95 m (0.125 ha) and were initially established between 2006 and 2007. They form part of an extensive network of permanent plots (609 in total) which covers the entire national park following a systematic segmented grid randomly superimposed onto the area (Lindsell and Klop 2013). Upon establishing the plots, each tree ≥30 cm in diameter was tagged, identified to species (or closest taxonomic unit) by a Sierra Leone Department of Forestry expert and its diameter (*D*_1_) recorded to the nearest 0.1 cm at a height of 1.3 m off the ground (or in the case of buttressed trees, at a known height above buttress). In addition, trees ≥10 cm in diameter were surveyed within a central subplot (radius 6.31 m; 0.0125 ha). Plots were recensused after an interval of 5–7 years, at which time stem diameters were remeasured (*D*_2_), tree deaths recorded, and any recruits tagged. Of the 2363 stems initially recorded, 189 died (median plot‐level mortality rate = 1.2% stems/year) and 257 trees recruited between the two census periods. A total of 167 unique tree species were recorded across the plot network, with 90% of stems identified to species and 94% at genus level [note that Talbot et al. (2014) suggest 80% of stems identified to genus as sufficient for productivity calculation].

#### Past logging activities

Prior to 1990, Gola was subjected to commercial selective logging, with timber extraction activities concentrated primarily in Gola South and in the western side of Gola Central (Lindsell and Klop 2013). By combining official logging offtake records (m^3^/ha of timber) with historical carbon stocking densities predating the commencement of logging activities in the park, Lindsell and Klop (2013) were able to map where logging had taken place within Gola. Here, we use this information to classify each surveyed plot as either logged (88 plots) or unlogged (54 plots) based on its location within the park. While the effects of logging on forest structure and function can vary substantially depending on logging practices (e.g., logging intensity, conventional versus reduced‐impact logging; Miller et al. 2011; West et al. 2014; Martin et al. 2015), here we focused on comparing logged versus unlogged plots as additional information on logging practices was unavailable for most of Gola.

#### Soil data

Soil samples from 48 of the 142 recensused plots were collected with the purpose of quantifying soil phosphorus (P), which has been shown to be a key driver of wood production across tropical forests (Cleveland et al. 2011; Quesada et al. 2012; Banin et al. 2014). In each plot, three 20‐cm‐deep soil cores were collected and then pooled into a single sample. Soil samples were oven dried at 60°C until constant weight was achieved, before being chemically processed in the laboratory (see Appendix S1 in Supporting Information for further details). Total soil P (mg/kg) was measured by inductively coupled plasma optical emission spectrometry (ICP‐OES).

Logistical constraints meant we were unable to collect soil samples from all field plots. Instead, we developed a regression model to estimate soil P for plots where no samples were collected. Specifically, soil P was modeled as a function of distance from streams (estimated in a GIS environment), terrain slope (measured using a clinometer), elevation (obtained from GPS data), and a random intercept term which allowed soil P levels to vary among plots clustered within transects (Tsui et al. 2004; Ferry et al. 2010). The model effectively captured variation in soil P (see Fig. S1 for details on model fit), and was used assign plots to one of three soil P classes (Benjamin Turner, personal communication): low (<300 mg P/kg; 36 plots), medium (300–500 mg P/kg; 61 plots), and high soil P (>500 mg P/kg; 45 plots).

### Quantifying aboveground wood production

Quantifying aboveground wood production (AWP) from permanent plot data presents a number of challenges, particularly in the context of tropical forests (Muller‐Landau et al. 2014; Talbot et al. 2014). Uncertainty in AWP estimates can arise from multiple sources, including (1) measurement errors resulting from imprecise field measurements (Rüger and Condit 2012) or changes in the position of measurement between censuses (e.g., due to the presence of buttress roots; Cushman et al. 2014); (2) missing information regarding the growth of trees that die and recruit between census periods (Malhi et al. 2004; Coomes et al. 2014; Talbot et al. 2014); (3) the use of allometric equations for scaling from diameter to aboveground biomass (Chave et al. 2014); and (4) the size of the area being sampled (Chave et al. 2004; Chambers et al. 2013). Here, we estimated AWP using the approach developed by Coomes et al. (2014), in which measured diameter increments are replaced with predicted growth estimates obtained from a statistical model in which tree growth is expressed as a function of trees size and competition for light. Below we describe the steps involved in estimating AWP and discuss how they aim to address the issues listed above. However, we acknowledge that the relatively small size of the permanent forest plots sampled in our study (0.125 ha) is a source of uncertainty which is likely to influence our AWP estimates, as the presence (or absence) of large trees within a plot will have a disproportionate impact on basal area and aboveground biomass estimates (Chave et al. 2004).

#### Step 1: data cleaning

Studies that rely on repeat census data to estimate tree growth rates routinely employ a number of screening procedures to minimize the presence of measurement errors which can otherwise bias productivity estimates. We started by calculating the annual diameter growth (*G*, in cm/year) of all trees that were alive at both census periods as (*D*_2_−*D*_1_)/Δ*t*, where Δ*t* is the time interval between censuses. Following the suggestions of Talbot et al. (2014), trees for which (1) *G* ≥ 4 cm/year or (2) whose diameter decreased by more than 0.5 cm between censuses were then excluded from the next step of the analysis (“modeling diameter growth”), as these values are considered extreme outliers arising from gross measurement errors (e.g., changes in the position of measurement between census periods or transcribing errors). Note that small negative *G* values were retained in the dataset to allow for stem shrinkage due to low hydrostatic pressure in the xylem during droughts (Talbot et al. 2014). At this stage, two plots which suffered particularly high mortality rates (>50% of stems died between the first and second census) were also excluded from all further analyses.

#### Step 2: modeling diameter growth

Annual diameter growth was modeled as a nonlinear function of tree size and competitive neighborhood (Coomes et al. 2012):

(1) $$G=\frac{\rho_{0}D^{\rho_{1}}\exp\left(\rho_{2}D\right)}{1+\rho_{3}\exp\left(\rho_{4}B_{L}\right)}$$

where *D* is a tree's diameter as measured during the first census (i.e., *D*_1_), *B*_L_ is the summed basal area of trees with a greater diameter than the target tree within the plot (described in this paragraph), and *ρ*0–*ρ*4 are parameters to be estimated from the data. The numerator of equation (1) is a modified power–law which defines the relationship between tree growth and size. This flexible function enables diameter growth to increase continuously with size, decelerate as trees become larger, or even peak and then decline as a function of initial diameter (Coomes et al. 2012). The denominator instead represents the effects of asymmetric competition for light on growth (Coomes and Allen 2007). The competitive effect of larger neighbors is captured by the competition term *B*_L_, which becomes progressively stronger as the density of trees larger than the focal tree increases (Coomes and Allen 2007; Cordonnier and Kunstler 2015). Equation (1) was chosen after extensive comparison with alternative growth functions (see Table S1) and was parameterized using nonlinear mixed‐effects models as implemented in R (3.0.1; R Core Development Team 2013) using the *nlme* library. To account for different growth trajectories among tree functional groups, estimated parameters were allowed to vary among tree genera (treated as a random effect in the model; Baraloto et al. 2012).

For each tree recorded during the first and second census period (including those that died or recruited between censuses), annual diameter growth estimates generated from equation (1) were then used to calculate the tree's diameter a year after its initial measurement (*t *+ 1). The advantage of this approach (compared to one where growth is imputed directly from field measurements) is (1) that measurement errors are absorbed by the model predictions and (2) that it provides a robust way to impute the growth of trees that died and recruited between each census based on their size and competitive status (Coomes et al. 2014; Talbot et al. 2014). However, it is important to note that this approach will also inevitably absorb part of the true variation in growth rates among trees. Furthermore, if the statistical model fails to adequately capture underlying patterns of tree growth (e.g., the relationship between tree growth and size), then our approach could potentially introduce systematic biases into AWP estimates. To rule out this possibility, we also calculated the diameter growth of all trees directly from field measurement using the protocol proposed by Talbot et al. (2014) and repeated all analyses with this alternative measure of growth (see Appendix S2 for details).

#### Step 3: converting from diameter to biomass growth

Diameter increments were converted to biomass growth by first calculating the aboveground biomass (AGB, in kg) of each tree at the time of the first census and at *t *+ 1, and then subtracting the two to obtain the annual biomass increment. AGB for both time periods was estimated using Chave et al. (2014) pan‐tropical biomass equation:

(2) $$\text{AGB}=0.0673\times {\left(D^{2}\times H\times \text{WD}\right)}^{0.976}$$

where a tree's biomass is expressed as a function of its diameter, height (*H*, in m), and wood density (WD, in g/cm^2^). WD values were obtained from a global database (Chave et al. 2009; Zanne et al. 2009), with stems being matched to the most resolved taxonomic unit possible following the suggestions of Lewis et al. (2009). *H* was estimated from *D* using the following Weibull function which we parameterized using height and diameter measurements made for 336 trees within Gola (see Appendix S2):

(3) $$H=79.9\times (1-\exp\left(-0.011\;D^{0.74}\right))$$

The above *H*–*D* equation showed considerably better fit to the data compared to other functional forms (e.g., power–law) as well as to published *H*–*D* equations for West African forests (e.g., Feldpausch et al. 2012; see Fig. S3).

#### Step 4: from individual tree growth to plot‐level AWP

The aboveground wood production (AWP, in MgC/ha year) of each plot was estimated by summing the annual biomass growth of all trees recorded during the first census. AWP was expressed in units of carbon by assuming a carbon concentration in woody tissues of 47% (Martin and Thomas 2011). Note that AWP estimates obtained using the statistical modeling approach described above closely match those calculated directly from field measurements (Fig. S4; Pearson's correlation coefficient = 0.92).

### Using structural equation models to identify key drivers of AWP

We used structural equation modeling (SEM) to test a conceptual model linking AWP to plot basal area (BA, in m^2^/ha), past logging, tree diversity, and soil P (Grace et al. 2010; Kline 2010). Central to the model is the relationship between AWP and BA. Basal area is intrinsically tied to aboveground biomass and stem packing density (e.g., Slik et al. 2010), both of which are key determinants of productivity in forests (Keeling and Phillips 2007; Coomes et al. 2014; Michaletz et al. 2014; Jenkins 2015). Specifically, densely packed stands tend to intercept more light and high‐biomass forests are generally dominated by larger, faster growing individual trees (Stephenson et al. 2014). An appealing property of BA is that it can be partitioned exactly into size and frequency components:

(4) $$\text{BA}=\pi /4\times {\left(\frac{\text{QMD}}{100}\right)}^{2}\times n_{\mathrm{stems}}$$

where *n*_stems_ is the stem density (number of stems/ha) and QMD is the quadratic mean stem diameter (in cm), which is calculated as $\sqrt{\left(\sum D^{2}\right)/n_{\mathrm{stems}}}$ (Curtis and Marshall 2000). We therefore chose to model BA as a composite variable of QMD and *n*_stems_ (Grace et al. 2010), which allowed us to explicitly attribute changes in BA to ones in either QMD and/or *n*_stems_. Specifically, we hypothesized that QMD would be lower in previously logged plots (Okuda et al. 2003; Bonnell et al. 2011; Osazuwa‐Peters et al. 2015) and on steep terrain (Clark and Clark 2000; Ferry et al. 2010; De Toledo et al. 2011), resulting in lower BA and thereby indirectly impacting AWP.

In addition to the pathway linking AWP to BA, we also modeled AWP as a function of tree diversity in order to test whether diverse plots are more productive than species‐poor ones (Chisholm et al. 2013; Vilà et al. 2013; Jucker et al. 2014). We quantified tree diversity as the exponential of the Shannon–Wiener index, which Jost (2006) defines as a measure of the “effective number of species”:

(5) $$\text{Effective}\;\text{no}\text{.}\;\text{species}=\exp\left(-\sum_{i=1}^{S}\frac{{\text{BA}}_{i}}{\text{BA}}\ln\left(\frac{{\text{BA}}_{i}}{\text{BA}}\right)\right)$$

where *S* is the number of unique species within a plot, BA_*i*_ is the basal area of species *i*, and BA is the total basal area of the plot. The advantage of this measure of diversity is that it accounts for differences in species' relative abundances while also providing a metric whose values are easily interpretable and directly relatable to species richness (Jost 2006). To account for the fact that a positive relationship between tree diversity and AWP might emerge simply because plots with a greater number of stems are also more species‐rich (Kadmon and Benjamini 2006), we included a pathway linking tree diversity to stem density in the SEM. Furthermore, we also tested whether tree diversity was impacted by past logging (e.g., Martin et al. 2013) and whether soil P content influences patters of tree diversity as has been suggested in the literature (Baltzer et al. 2005; Russo et al. 2008; Coomes et al. 2009; Fortunel et al. 2014). Lastly, based on the assumption that productivity in tropical forests is strongly limited by soil phosphorus content (Vitousek et al. 2010; Cleveland et al. 2011; Quesada et al. 2012; Banin et al. 2014), we included a direct pathway between soil P and AWP in the model.

#### Model fitting and evaluation

SEMs were implemented using the *lavaan* R package (Rosseel 2012). To normalize model residuals, AWP, *n*_stems_, and QMD were log‐transformed prior to model fitting (Grace et al. 2010), while Soil P (ordinal categorical variable) and logging (binary covariate) were both treated as numeric predictors in the model (Rosseel 2012; Zhang and Chen 2015). Following the suggestions of Kline (2010) the fit of the SEM was evaluated based on the following criteria: chi‐square test and associated *P* value (where *P* > 0.05 indicates that sample and observed covariance matrices are statistically indistinguishable), the root mean square error of approximation (RMSEA; target value < 0.05), the comparative fit index (CFI; target value > 0.90), and the standardized root mean square residual (SRMR; target value < 0.10). Lastly, standardized path coefficients (and associated *P* values) were calculated for individual pathways in the model in order to assess the relative contribution of each predictor to patterns of AWP (Grace and Bollen 2005).

## Results

**Figure 2:**
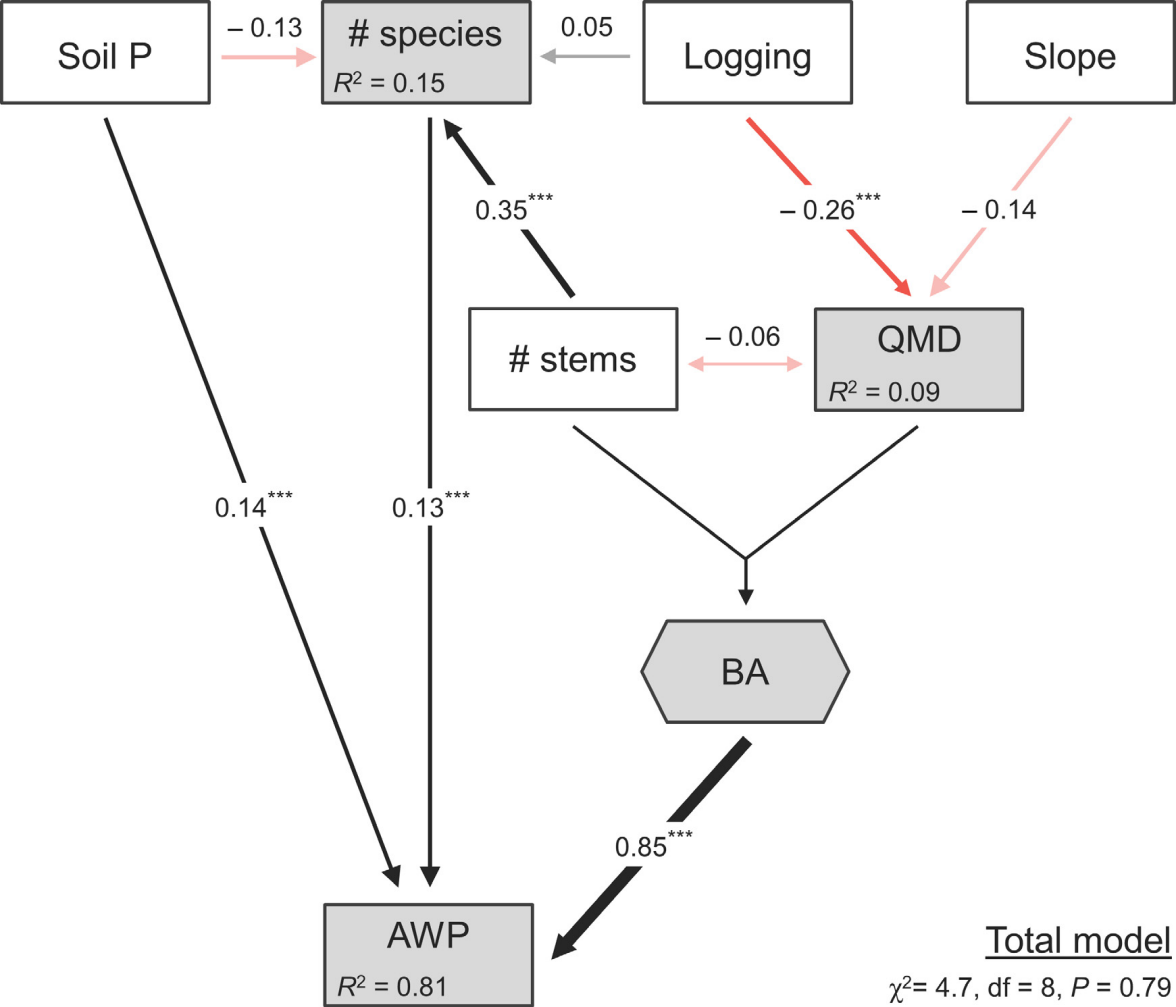
Structural equation model relating variation in aboveground wood production (AWP) to basal area (BA), effective number of tree species (# species), and soil phosphorus (P). BA was modeled as a composite variable of quadratic mean diameter (QMD) and stem density (# stems), which together determine BA exactly [see eq. (4) in the text]. Tree diversity is expressed as a function of stem density, soil P, and past logging, which in conjunction with terrain slope is also assumed to influence QMD. Exogenous variables are represented by white boxes, while endogenous variables are shaded in gray. The width of the arrows reflects the strength of the pathway and is proportional to the standardized path coefficient (which is reported for each pathway). Black arrows denote positive relationships, while red arrows correspond to negative ones. Note that a bidirectional arrow is used to report the estimated covariance between stem density and QMD. Asterisks denote significance levels of the pathways in the model (*P < 0.05; **P < 0.01; ***P < 0.001; nonsignificant pathways are represented by semitransparent arrows). *R* ^2^ values are reported for each endogenous variable and model fit statistics are given in the bottom right‐hand corner.

Across the network of permanent forest plots AWP ranged between 0.53 and 4.31 Mg C/ha/year (mean AWP = 1.80 Mg C/ha/year). The SEM provided a good fit to the data (*χ* ^2^ = 4.7, df = 8, *P* = 0.79; RMSEA = 0.001; CFI = 0.999; SRMR = 0.027), and as a whole explained 81% of the variation in AWP among plots (Fig. 2).

**Figure 3:**
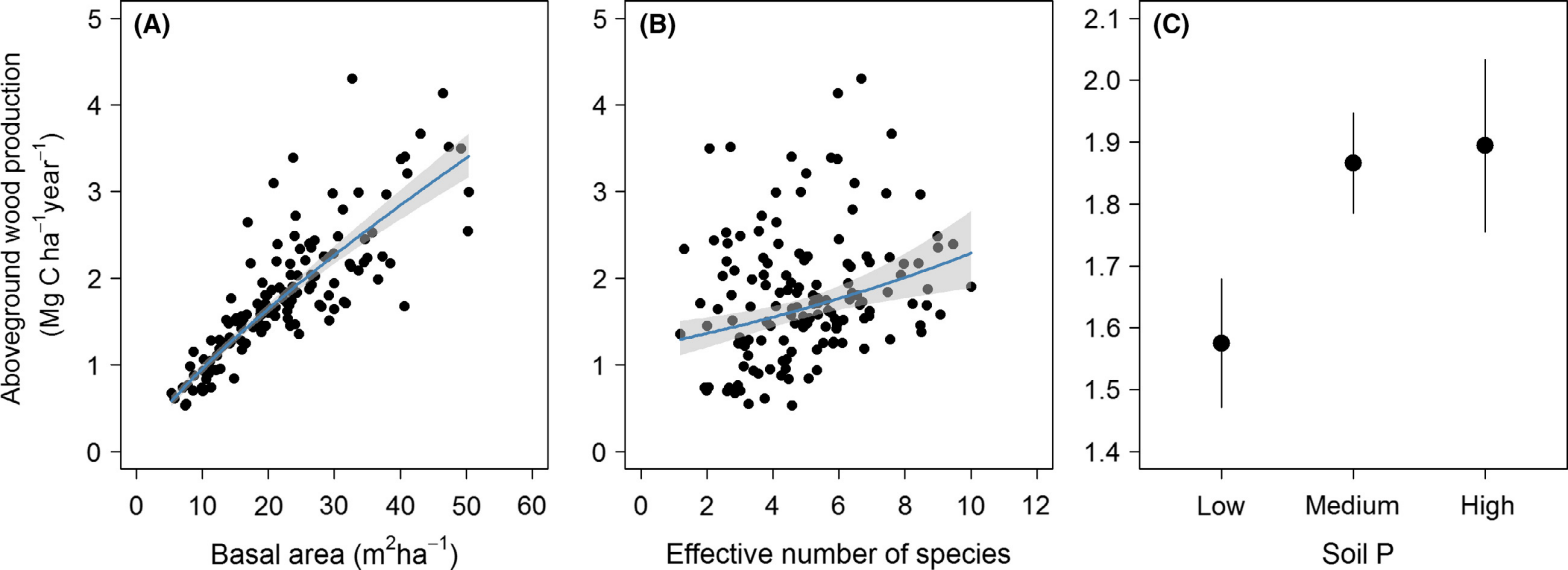
Relationship between aboveground wood production (AWP) and (A) plot basal area, (B) effective number of tree species, and (C) soil P. Fitted regression curves (back‐transformed from logarithmic scale) with 95% confidence intervals shaded in gray are shown for panels (A–B). Panel (C) shows the variation in the mean AWP (±1 SE) among the soil P classes. Note that the scale of the y‐axis in panel (C) does not match the previous two panels.

**Figure 4:**
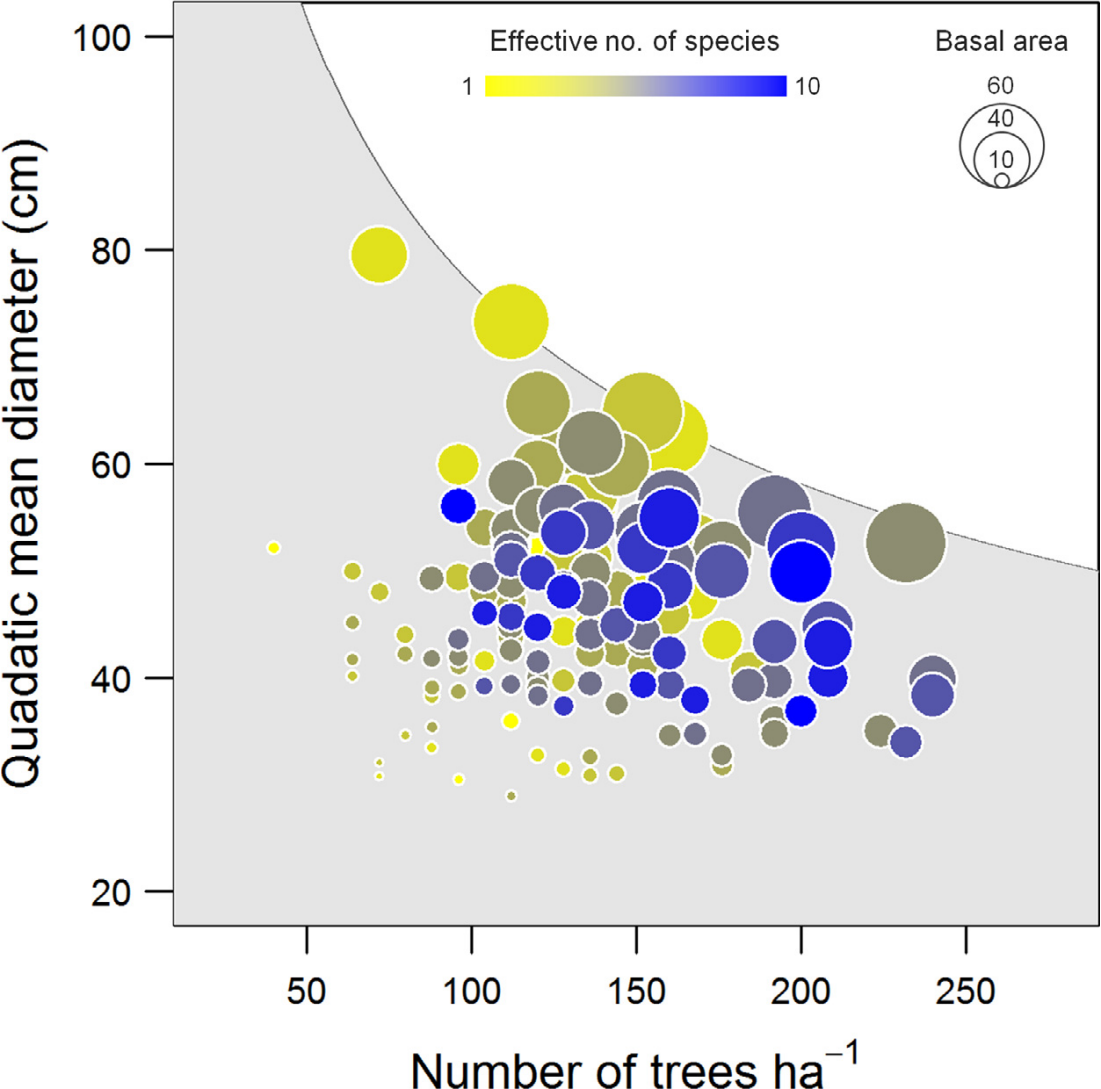
Relationship between quadratic mean stem diameter and number of stems per hectare. The size of the points reflects the basal area of the plot (m^2^/ha), while the shading is determined by the effective number of tree species. For visual purposes only, a self‐thinning curve is shown in gray and highlights the inherent trade‐off between the number and mean size of trees in forests. The shape of the self‐thinning curve was determined by fitting a regression (on log–log scale) to the 99th quantile of the data using the *quantreg* package in R. Note that while stem density and basal area have been scaled up to a per‐hectare basis, the effective number of species refers to plot‐level measurements (i.e., 0.125 ha) as diversity does not scale linearly with area.

**Figure 5:**
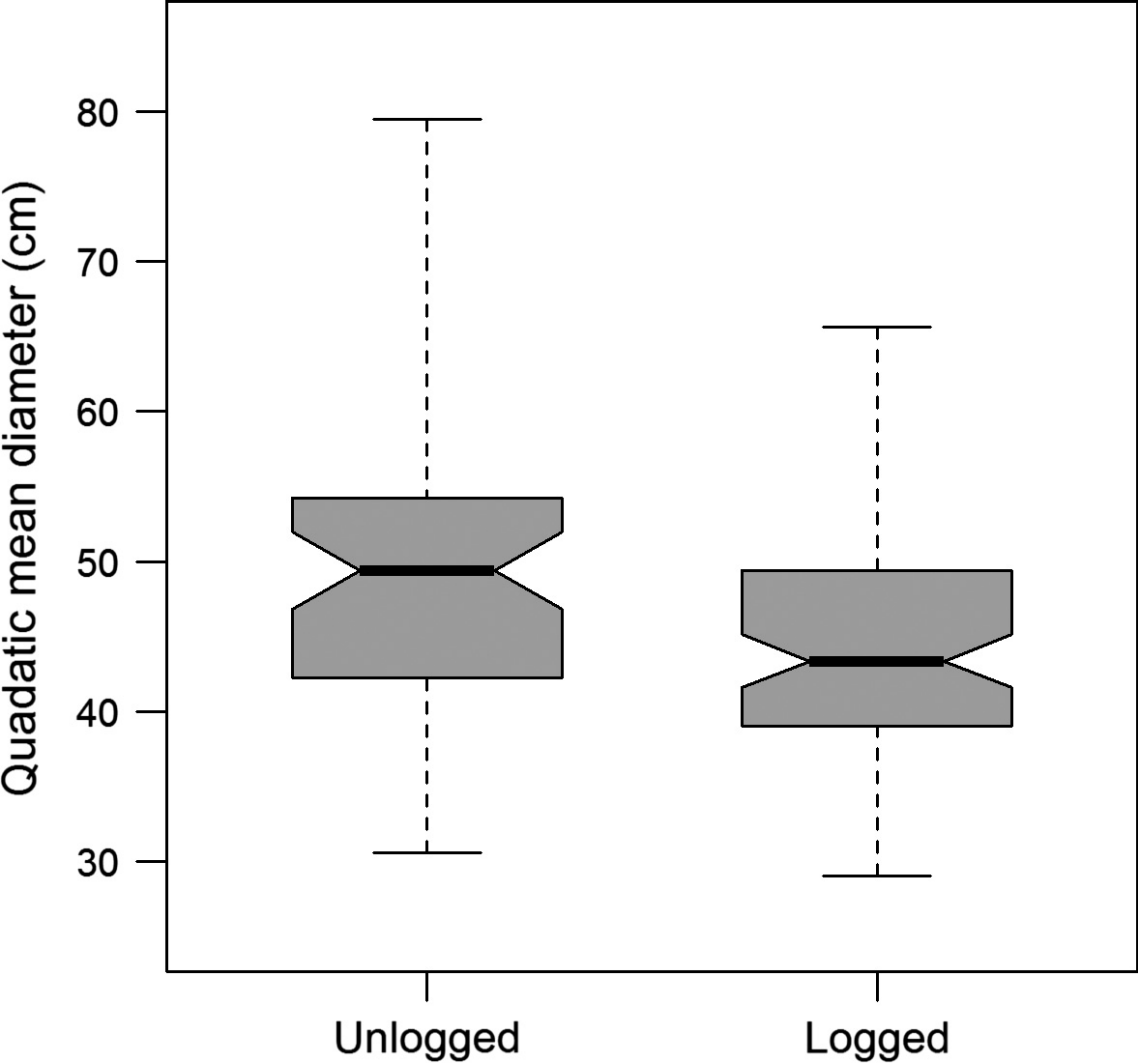
Mean quadratic stem diameter of unlogged and selectively logged forest plots. Notches in the boxplots indicate the 95% confidence intervals of the medians for the two groups. Nonoverlapping notches are strong evidence that medians differ between groups.

With the exception of terrain slope, all predictors included in the SEM contributed significantly to shaping patterns of AWP (Fig. 2). The single strongest determinant of AWP was BA (Fig. 3A), with the relationship between the two being best described by a power–law function with an exponent of 0.76 (95% CI = ±0.11). In addition to basal area, both tree diversity and soil P also contributed to promoting AWP (Fig. 3B–C), with the direct effect of the two drivers being comparable in magnitude (Fig. 2). In particular, the positive effect of tree diversity on AWP emerged even after having controlled for the strong dependence of tree diversity on stem density (Fig. 2). Diverse plots generally had greater stem densities (for a given QMD) compared to species‐poor ones (Fig. 4), resulting in a positive relationship between tree diversity and both BA and AWP (Figs. 3B and S5). Lastly, historical logging indirectly impacted AWP by causing a reduction in QMD (Fig. 5), and thereby BA. On average, QMD was 5 cm lower in previously logged plots compared to old‐growth forests, which equated to a loss in AWP of 0.27 Mg C/ha/year (95% CI = ±0.19 Mg C/ha/year). In contrast, we found no support for the idea that historical logging negatively impacted tree diversity, and only a weak negative association between soil P and tree diversity (*P* = 0.09; Fig. 2).

Quantitatively very similar results to those presented above were found when the SEM was fit to AWP estimates obtained directly from field measurements (as opposed to ones derived using the statistical modeling approach described in Materials and Methods; see Fig. S5). In addition, a complementary analysis of the data using multiple regression in place of SEMs revealed that results were robust to the choice of analytical tool (see Appendix S2).

## Discussion

Rates of aboveground wood production varied considerably across Gola Rainforest National Park, with forest plots exhibiting nearly an order of magnitude difference in AWP between the least and most productive stands. Structural equation modeling enabled us to identify a set of key drivers that, either directly or indirectly, contributed to shaping patterns of AWP across Gola (Fig. 2). We found that basal area – which reflects the frequency and mean size of stems in a plot – was central to explaining variation in AWP (Fig. 3A). In addition to the strong positive relationship between AWP and basal area, tree species diversity and soil P content also contributed to promoting AWP (Fig. 3B–C). By contrast, historical logging negatively impacted AWP, an effect which was mediated through the removal of large trees which play a central role in driving carbon sequestration in forests (Fig. 5).

### Basal area as a key determinant of AWP

Stand basal area emerged as the strongest determinant of AWP across Gola, with plots characterized by high basal areas also exhibiting the highest rates of AWP (Figs. 2 and 3A). This strongly positive relationship between AWP and basal area matches the reports of numerous papers which have shown that productivity is strongly coupled with aboveground biomass in forest systems (Keeling and Phillips 2007; Michaletz et al. 2014; Jenkins 2015). Basal area effectively captures the degree to which trees pack and utilize space aboveground (Jucker et al. 2015), which in turn is a key determinant of light interception and growth at the stand level (Coomes et al. 2014). As discussed previously, basal area is a product of both the size and number of stems within a stand. In this respect, our results seem to suggest that stands with high QMD were generally more productive than ones dominated by a large number of smaller stems (Fig. S6), highlighting the key role played by large trees in determining rates of AWP in forests (Slik et al. 2013; Stephenson et al. 2014; Bastin et al. 2015).

Our results suggest that while the relationship between AWP and basal area does begin to saturate at high packing densities (i.e., the exponent of the power–law relationship is <1; see Fig. 3A), this saturation effect is rather weak. In fact, across Gola we found no evidence of AWP declining in plots with high basal areas which are generally dominated by larger – and presumably older – trees (Ryan et al. 1997; Magnani et al. 2000). One explanation for this lack of age‐related decline in productivity could well be that large parts of Gola are dominated by relatively young secondary forest which is still recovering from past disturbance (Lindsell and Klop 2013), as evidenced by the fact that many of the surveyed plots have relatively low basal areas (Lewis et al. 2013; Fig. 3A). In addition to this, the fact that AWP does not decline at high packing densities may also reflect the fact that as basal area accumulates during stand development, forests optimize the structure and photosynthetic physiology of their canopies in a manner which maintains high rates of carbon assimilation (Hardiman et al. 2011, 2013; Coomes et al. 2012). While this last hypothesis is something we are unable to test directly with the current dataset, further work attempting to understand how forests are able to maintain high rates of AWP in the later stages of stand development is needed.

### Tree species diversity promotes AWP

Across Gola forest we found that diverse plots were generally more productive than species‐poor ones (Fig. 3B). This finding matches those of a growing number of studies reporting positive relationships between diversity and productivity in forests (Paquette and Messier 2011; Vilà et al. 2013; Jucker et al. 2014), including in the context of tropical forests (Chisholm et al. 2013; Lasky et al. 2014). The fact that diversity generally seems to promote AWP may be the result of niche complementarity, whereby combining species with complementary ecological strategies enables individuals to compete less fiercely and diverse communities to use resources more efficiently (Loreau and Hector 2001). Additionally, reduced pest and pathogen loads in diverse forest patches could also contribute to the positive relationship between tree diversity and AWP which we observe (Jactel and Brockerhoff 2007). Interestingly, because the relationship between diversity and productivity has generally been found to be a saturating one (i.e., at a certain point adding new species to a community no longer results in a gain in productivity; Cardinale et al. 2006), there was reason to believe that diversity effects on AWP should be relatively weak in hyperdiverse tropical forests (Lasky et al. 2014). However, especially at relatively small spatial scales such as those of the Gola forest plots (Chisholm et al. 2013), our results suggest that tree diversity can play an important role in driving AWP even in the context of tropical forests (Poorter et al. 2015).

In addition to directly enhancing the growth of individual trees, another pathway through which diversity can promote AWP is by enabling trees to pack more densely in space (Pretzsch 2014; Sapijanskas et al. 2014; Jucker et al. 2015). For instance, Chisholm et al. (2013) found that across the tropics diverse forest patches generally have greater aboveground biomass stocks compared to ones with fewer tree species, which as our analysis shows has important implications for AWP. In our study, we found a strong degree of covariation between tree diversity and stem density (Fig. 2). While this positive association primarily reflects a typical species accumulation curve (Kadmon and Benjamini 2006), it is likely that at least in part covariation between tree diversity and stem density is determined by the fact that diverse stands are able to pack more stems in a given area.

### Soil nutrient availability modulates AWP

Soil phosphorus content emerged as strong determinant of AWP across Gola (Fig. 3C), highlighting the important role played by soil nutrients in driving variation in productivity in tropical forests (Cleveland et al. 2011; Quesada et al. 2012; Banin et al. 2014). Previous work in the Amazon and in tropical forests of Southeast Asia also found productivity to increase markedly in response to total soil P content (Quesada et al. 2012; Banin et al. 2014), supporting the view that lowland tropical forests are P‐limited (Whitmore 1990; Vitousek et al. 2010). While nitrogen‐fixing microorganisms generally help maintain high nitrogen concentrations in tropical forest soils (Hedin et al. 2009), soil P is primarily supplied through weathering of mineral bedrock and in the lowland tropics is rapidly leached from the mineral subsoil, resulting in high soil N:P ratios and P‐depleted soils (Vitousek et al. 2010). Soil P content can directly influence forest productivity by affecting the ecophysiology and growth of individual tree species. For instance, leaf nutrient concentrations have been shown to be strongly tied to those in the soil (Tanner et al. 1998; Townsend et al. 2007), which in turn has major implications for species' photosynthetic rates and carbon uptake at stand level (Mercado et al. 2011; Reich 2012).

In addition to directly limiting tree growth, soil nutrients can also impact forest productivity indirectly as a result of species filtering along edaphic gradients (Russo et al. 2005; Reich 2014). Many tropical tree species have been shown to exhibit clear habitat preferences which relate directly to soil type (Russo et al. 2005; John et al. 2007; Condit et al. 2013). In particular, nutrient‐poor soils tend to be dominated by species with conservative strategies, having leaf, root, and architectural traits that maximize survival (Baltzer et al. 2005; Poorter and Bongers 2006; Sterck et al. 2006, 2011; Russo et al. 2008; Gourlet‐Fleury et al. 2011; Holdaway et al. 2011; Fortunel et al. 2014). In contrast, it has been hypothesized that nutrient‐rich soils can support a wider range of species, including ones characterized by resource‐acquisitive traits which rely on fast growth of relatively inexpensive plant tissues to escape shaded understories (Sterck et al. 2006; Russo et al. 2008; Coomes et al. 2009; Fortunel et al. 2014; Reich 2014). However, in contrast to expectations we found no clear influence of soil P content on tree species diversity across Gola forest (Fig. 2). Further work on how soil nutrients contribute to shaping patterns of tree species diversity and composition in the African tropics is needed, as most studies to date have taken place in the Neotropics and Southeast Asia.

### Long‐term impacts of logging on AWP

Logging activities can impact forest productivity in numerous ways, such as damaging trees and altering canopy structure (Okuda et al. 2003; Asner et al. 2004; Blanc et al. 2009; Martin et al. 2013; West et al. 2014), promoting the establishment of lianas (Schnitzer and Bongers 2011; Durán et al. 2013) and as a result of soil erosion (Chazdon 2003). Across Gola forest we found that historical logging activities had a long‐lasting impact on the mean size of trees within plots (Fig. 5). In turn, the targeted removal of large trees through selective logging operations negatively affected AWP (Fig. 2), further highlighting the key role played by large trees in driving carbon sequestration rates in forests (Slik et al. 2013; Stephenson et al. 2014).

Our results suggest that the effects of selective logging on tree size distributions – and, indirectly, on productivity – can persist for decades after logging activities cease, which matches a number of other reports in the literature (Okuda et al. 2003; Bonnell et al. 2011; Lindsell and Klop 2013; Martin et al. 2013; Osazuwa‐Peters et al. 2015). Supporting these findings, Kent et al. (2015) recently used airborne LiDAR imagery covering a vast swathe of Gola forest to show that these same logging operations left a clear and detectable fingerprint on the vertical structure of the forest canopy. Conversely, our results do not suggest that logging activities have had a long‐lasting impact on tree diversity in Gola (Fig. 2), which contrasts with reports highlighting how diversity often takes longer to recover from the logging than aboveground carbon pools (Martin et al. 2013). One possible explanation for the limited impact of logging on tree diversity is that logging operations in Gola were highly selective, focusing only on limited number of commercially valuable timber species (Gourlet‐Fleury et al. 2013; Lindsell and Klop 2013).

## Conclusions

Despite the fact that only a small fraction of the carbon fixed by forest canopies is allocated to wood, wood production plays a critical role in determining the long‐term dynamics of carbon in forests. Consequently, understanding what factors are important in controlling rates of AWP in forests has major implications for projecting the terrestrial carbon cycle into an increasingly uncertain future. This is particularly true in the context of tropical forests, which store much of the terrestrial carbon and yet remain relatively understudied. Here, we provide what to our knowledge is one of the few accounts of wood production in tropical forests of West Africa. Our results highlight how AWP can vary substantially even within the relatively small confines of Gola Rainforest National Park, and show how multiple biotic and abiotic drivers – including the size, number, and diversity of trees as well as the availability of soil nutrients – come together to shape rates of AWP. Within this context, disturbance associated with human activities such as logging can have a long‐lasting impact on a forest's ability to sequester and store carbon, further highlighting the importance of safeguarding what remains of old‐growth tropical forests.

## Data Accessibility

AWP data: uploaded as online supporting information.

## Conflict of Interest

None declared.

## Supporting information


**Appendix S1**. Soil data.
**Appendix S2**. Estimating AWP from permanent plot data.
**Appendix S3**. Correlations among model predictors.

**Appendix S4**. AWP data.

## References

[ece32175-bib-0001] Asner, G. P. , M. Keller , R. Pereira , J. C. Zweede , and J. N. M. Silva . 2004 Canopy damage and recovery after selective logging in Amazonia: field and satellite studies. Ecol. Appl. 14:280–298.

[ece32175-bib-0002] Asner, G. P. , T. K. Rudel , T. M. Aide , R. Defries , and R. Emerson . 2009 A contemporary assessment of change in humid tropical forests. Conserv. Biol. 23:1386–1395. 20078639 10.1111/j.1523-1739.2009.01333.x

[ece32175-bib-0003] Baker, T. R. , O. L. Phillips , Y. Malhi , S. Almeida , L. P. Arroyo , A. Di Fiore , et al. 2004 Increasing biomass in Amazonian forest plots. Philos. Trans. R. Soc. B 359:353–365. 10.1098/rstb.2003.1422 PMC1693327 15212090

[ece32175-bib-0004] Baltzer, J. L. , S. C. Thomas , R. Nilus , and D. F. R. P. Burslem . 2005 Edaphic specialization in tropical trees: physiological correlates and responses to reciprocal transplantation. Ecology 86:3063–3077.

[ece32175-bib-0005] Banin, L. , S. L. Lewis , G. Lopez‐Gonzalez , T. R. Baker , C. A. Quesada , K. J. Chao , et al. 2014 Tropical forest wood production: a cross‐continental comparison. J. Ecol. 102:1025–1037.

[ece32175-bib-0006] Baraloto, C. , O. J. Hardy , C. E. T. Paine , K. G. Dexter , C. Cruaud , L. T. Dunning , et al. 2012 Using functional traits and phylogenetic trees to examine the assembly of tropical tree communities. J. Ecol. 100:690–701.

[ece32175-bib-0007] Bastin, J.‐F. , N. Barbier , M. Réjou‐Méchain , A. Fayolle , S. Gourlet‐Fleury , D. Maniatis , et al. 2015 Seeing Central African forests through their largest trees. Sci. Rep. 5:13156. 26279193 10.1038/srep13156 PMC4538397

[ece32175-bib-0008] Blanc, L. , M. Echard , B. Herault , D. Bonal , E. Marcon , J. Chave , et al. 2009 Dynamics of aboveground carbon stocks in a selectively logged tropical forest. Ecol. Appl. 19:1397–1404. 19769089 10.1890/08-1572.1

[ece32175-bib-0009] Boisvenue, C. , and S. W. Running . 2006 Impacts of climate change on natural forest productivity – evidence since the middle of the 20th century. Glob. Change Biol. 12:862–882.

[ece32175-bib-0010] Bonnell, T. R. , R. Reyna‐Hurtado , and C. A. Chapman . 2011 Post‐logging recovery time is longer than expected in an East African tropical forest. For. Ecol. Manage. 261:855–864.

[ece32175-bib-0011] Brienen, R. J. W. , O. L. Phillips , T. R. Feldpausch , E. Gloor , T. R. Baker , J. Lloyd , et al. 2015 Long‐term decline of the Amazon carbon sink. Nature 519:344–348. 25788097 10.1038/nature14283

[ece32175-bib-0012] Cardinale, B. J. , D. S. Srivastava , J. E. Duffy , J. P. Wright , A. L. Downing , M. Sankaran , et al. 2006 Effects of biodiversity on the functioning of trophic groups and ecosystems. Nature 443:989–992. 17066035 10.1038/nature05202

[ece32175-bib-0013] Chambers, J. Q. , R. I. Negron‐Juarez , D. M. Marra , A. Di Vittorio , J. Tews , D. Roberts , et al. 2013 The steady‐state mosaic of disturbance and succession across an old‐growth Central Amazon forest landscape. Proc. Natl Acad. Sci. 110:3949–3954. 23359707 10.1073/pnas.1202894110 PMC3593828

[ece32175-bib-0014] Chave, J. , R. Condit , S. Aguilar , A. Hernandez , S. Lao , and R. Perez . 2004 Error propagation and scaling for tropical forest biomass estimates. Philos. Trans. R. Soc. B 359:409–420. 10.1098/rstb.2003.1425 PMC1693335 15212093

[ece32175-bib-0015] Chave, J. , D. A. Coomes , S. Jansen , S. L. Lewis , N. G. Swenson , and A. E. Zanne . 2009 Towards a worldwide wood economics spectrum. Ecol. Lett. 12:351–366. 19243406 10.1111/j.1461-0248.2009.01285.x

[ece32175-bib-0016] Chave, J. , M. Réjou‐Méchain , A. Búrquez , E. Chidumayo , M. S. Colgan , W. B. C. Delitti , et al. 2014 Improved allometric models to estimate the aboveground biomass of tropical trees. Glob. Change Biol. 20:3177–3190. 10.1111/gcb.12629 24817483

[ece32175-bib-0017] Chazdon, R. L. 2003 Tropical forest recovery: legacies of human impact and natural disturbances. Perspect. Plant Ecol. Evol. Syst. 6:51–71.

[ece32175-bib-0018] Chisholm, R. A. , H. C. Muller‐Landau , K. Abdul Rahman , D. P. Bebber , Y. Bin , S. A. Bohlman , et al. 2013 Scale‐dependent relationships between tree species richness and ecosystem function in forests. J. Ecol. 101:1214–1224.

[ece32175-bib-0019] Clark, D. B. , and D. A. Clark . 2000 Landscape‐scale variation in forest structure and biomass in a tropical rain forest. For. Ecol. Manage. 137:185–198.

[ece32175-bib-0020] Clark, D. A. , S. C. Piper , C. D. Keeling , and D. B. Clark . 2003 Tropical rain forest tree growth and atmospheric carbon dynamics linked to interannual temperature variation during 1984‐2000. Proc. Natl Acad. Sci. 100:5852–5857. 12719545 10.1073/pnas.0935903100 PMC156290

[ece32175-bib-0021] Cleveland, C. C. , A. R. Townsend , P. Taylor , S. Alvarez‐Clare , M. M. C. Bustamante , G. Chuyong , et al. 2011 Relationships among net primary productivity, nutrients and climate in tropical rain forest: a pan‐tropical analysis. Ecol. Lett. 14:939–947. 21749602 10.1111/j.1461-0248.2011.01658.x

[ece32175-bib-0022] Condit, R. , B. M. J. Engelbrecht , D. Pino , R. Pérez , and B. L. Turner . 2013 Species distributions in response to individual soil nutrients and seasonal drought across a community of tropical trees. Proc. Natl Acad. Sci. 110:5064–5068. 23440213 10.1073/pnas.1218042110 PMC3612601

[ece32175-bib-0023] Coomes, D. A. , and R. B. Allen . 2007 Effects of size, competition and altitude on tree growth. J. Ecol. 95:1084–1097.

[ece32175-bib-0024] Coomes, D. A. , G. Kunstler , C. D. Canham , and E. Wright . 2009 A greater range of shade‐tolerance niches in nutrient‐rich forests: an explanation for positive richness‐productivity relationships? J. Ecol. 97:705–717.

[ece32175-bib-0025] Coomes, D. A. , R. J. Holdaway , R. K. Kobe , E. R. Lines , and R. B. Allen . 2012 A general integrative framework for modelling woody biomass production and carbon sequestration rates in forests. J. Ecol. 100:42–64.

[ece32175-bib-0026] Coomes, D. A. , O. Flores , R. Holdaway , T. Jucker , E. R. Lines , and M. C. Vanderwel . 2014 Wood production response to climate change will depend critically on forest composition and structure. Glob. Change Biol. 20:3632–3645. 10.1111/gcb.12622 24771558

[ece32175-bib-0027] Cordonnier, T. , and G. Kunstler . 2015 The Gini index brings asymmetric competition to light. Perspect. Plant Ecol. Evol. Syst. 17:107–115.

[ece32175-bib-0028] Curtis, R. P. , and D. D. Marshall . 2000 Why quadratic mean diameter? West. J. Appl. Forestry 15:137–139.

[ece32175-bib-0029] Cushman, K. C. , H. C. Muller‐Landau , R. S. Condit , and S. P. Hubbell . 2014 Improving estimates of biomass change in buttressed trees using tree taper models. Methods Ecol. Evol. 5:573–582.

[ece32175-bib-0030] De Toledo, J. J. , W. E. Magnusson , C. V. Castilho , and H. E. M. Nascimento . 2011 How much variation in tree mortality is predicted by soil and topography in Central Amazonia? For. Ecol. Manage. 262:331–338.

[ece32175-bib-0031] Dong, S. X. , S. J. Davies , P. S. Ashton , S. Bunyavejchewin , M. N. N. Supardi , A. R. Kassim , et al. 2012 Variability in solar radiation and temperature explains observed patterns and trends in tree growth rates across four tropical forests. Proc. R. Soc. B 279:3923–3931. 10.1098/rspb.2012.1124 PMC3427576 22833269

[ece32175-bib-0032] Durán, S. M. , E. Gianoli , and S. M. Dura . 2013 Carbon stocks in tropical forests decrease with liana density. Biol. Lett. 9:20130301. 23784930 10.1098/rsbl.2013.0301 PMC3730642

[ece32175-bib-0033] Feeley, K. J. , S. Joseph Wright , M. N. Nur Supardi , A. R. Kassim , and S. J. Davies . 2007 Decelerating growth in tropical forest trees. Ecol. Lett. 10:461–469. 17498145 10.1111/j.1461-0248.2007.01033.x

[ece32175-bib-0034] Feldpausch, T. R. , J. Lloyd , S. L. Lewis , R. J. W. Brienen , M. Gloor , A. Monteagudo Mendoza , et al. 2012 Tree height integrated into pantropical forest biomass estimates. Biogeosciences 9:3381–3403.

[ece32175-bib-0035] Ferry, B. , F. Morneau , J. D. Bontemps , L. Blanc , and V. Freycon . 2010 Higher treefall rates on slopes and waterlogged soils result in lower stand biomass and productivity in a tropical rain forest. J. Ecol. 98:106–116.

[ece32175-bib-0036] Fortunel, C. , C. E. T. Paine , P. V. A. Fine , N. J. B. Kraft , and C. Baraloto . 2014 Environmental factors predict community functional composition in Amazonian forests. J. Ecol. 102:145–155.

[ece32175-bib-0037] Gourlet‐Fleury, S. , V. Rossi , M. Rejou‐Mechain , V. Freycon , A. Fayolle , L. Saint‐André , et al. 2011 Environmental filtering of dense‐wooded species controls above‐ground biomass stored in African moist forests. J. Ecol. 99:981–990.

[ece32175-bib-0038] Gourlet‐Fleury, S. , D. Beina , A. Fayolle , D. Y. Ouédraogo , F. Mortier , F. Bénédet , et al. 2013 Silvicultural disturbance has little impact on tree species diversity in a Central African moist forest. For. Ecol. Manage. 304:322–332.

[ece32175-bib-0039] Grace, J. B. , and K. A. Bollen . 2005 Interpreting the results from multiple regression and structural equation models. Bull. Ecol. Soc. Am. 86:283–295.

[ece32175-bib-0040] Grace, J. B. , T. M. Anderson , H. Olff , and S. M. Scheiner . 2010 On the specification of structural equation models for ecological systems. Ecol. Monogr. 80:67–87.

[ece32175-bib-0041] Hardiman, B. S. , G. Bohrer , C. M. Gough , C. S. Vogel , and P. S. Curtis . 2011 The role of canopy structural complexity in wood net primary production of a maturing northern deciduous forest. Ecology 92:1818–1827. 21939078 10.1890/10-2192.1

[ece32175-bib-0042] Hardiman, B. S. , C. M. Gough , A. Halperin , K. L. Hofmeister , L. E. Nave , G. Bohrer , et al. 2013 Maintaining high rates of carbon storage in old forests: a mechanism linking canopy structure to forest function. For. Ecol. Manage. 298:111–119.

[ece32175-bib-0043] Hedin, L. O. , E. N. J. Brookshire , D. N. L. Menge , and A. R. Barron . 2009 The nitrogen paradox in tropical forest ecosystems. Annu. Rev. Ecol. Evol. Syst. 40:613–635.

[ece32175-bib-0044] Holdaway, R. J. , S. J. Richardson , I. A. Dickie , D. A. Peltzer , and D. A. Coomes . 2011 Species‐ and community‐level patterns in fine root traits along a 120000‐year soil chronosequence in temperate rain forest. J. Ecol. 99:954–963.

[ece32175-bib-0045] Jactel, H. , and E. G. Brockerhoff . 2007 Tree diversity reduces herbivory by forest insects. Ecol. Lett. 10:835–848. 17663717 10.1111/j.1461-0248.2007.01073.x

[ece32175-bib-0046] Jenkins, D. G. 2015 Estimating ecological production from biomass. Ecosphere 6:49.

[ece32175-bib-0047] John, R. , J. W. Dalling , K. E. Harms , J. B. Yavitt , R. F. Stallard , M. Mirabello , et al. 2007 Soil nutrients influence spatial distributions of tropical tree species. Proc. Natl Acad. Sci. 104:864–869. 17215353 10.1073/pnas.0604666104 PMC1783405

[ece32175-bib-0048] Jost, L. 2006 Entropy and diversity. Oikos 113:363–375.

[ece32175-bib-0049] Jucker, T. , O. Bouriaud , D. Avăcăriei , and D. A. Coomes . 2014 Stabilizing effects of diversity on aboveground wood production in forest ecosystems: linking patterns and processes. Ecol. Lett. 17:1560–1569. 25308256 10.1111/ele.12382

[ece32175-bib-0050] Jucker, T. , O. Bouriaud , and D. A. Coomes . 2015 Crown plasticity enables trees to optimize canopy packing in mixed‐species forests. Funct. Ecol. 29:1078–1086.

[ece32175-bib-0051] Kadmon, R. , and Y. Benjamini . 2006 Effects of productivity and disturbance on species richness: a neutral model. Am. Nat. 167:939–946. 16685638 10.1086/504602

[ece32175-bib-0052] Keeling, H. C. , and O. L. Phillips . 2007 The global relationship between forest productivity and biomass. Glob. Ecol. Biogeogr. 16:618–631.

[ece32175-bib-0053] Kent, R. , J. A. Lindsell , G. V. Laurin , R. Valentini , and D. A. Coomes . 2015 Airborne LiDAR detects selectively logged tropical forest even in an advanced stage of recovery. Remote Sens. 7:8348–8367.

[ece32175-bib-0054] Kline, R. B. 2010 Principles and practice of structural equation modeling. Guilford Press, New York.

[ece32175-bib-0055] Klop, E. , J. A. Lindsell , and A. Siaka . 2008 Biodiversity of Gola forest, Sierra Leone. Royal Society for the Protection of Birds, Conservation Society of Sierra Leone, and Government of Sierra Leone, Sandy, UK, and Freetown, Sierra Leone.

[ece32175-bib-0056] Lasky, J. R. , M. Uriarte , V. K. Boukili , D. L. Erickson , W. J. Kress , and R. L. Chazdon . 2014 The relationship between tree biodiversity and biomass dynamics changes with tropical forest succession. Ecol. Lett. 17:1158–1167. 24986005 10.1111/ele.12322

[ece32175-bib-0057] Laurance, W. F. 1999 Effects on the tropical deforestation crisis. Biol. Conserv. 91:109–117.

[ece32175-bib-0058] Lewis, S. L. , G. Lopez‐Gonzalez , B. Sonké , K. Affum‐Baffoe , T. R. Baker , L. O. Ojo , et al. 2009 Increasing carbon storage in intact African tropical forests. Nature 457:1003–1006. 19225523 10.1038/nature07771

[ece32175-bib-0059] Lewis, S. L. , B. Sonké , T. Sunderland , S. K. Begne , G. Lopez‐Gonzalez , G. M. F. van der Heijden , et al. 2013 Above‐ground biomass and structure of 260 African tropical forests. Philos. Trans. R. Soc. B 368:20120295. 10.1098/rstb.2012.0295 PMC3720018 23878327

[ece32175-bib-0060] Lindsell, J. A. , and E. Klop . 2013 Spatial and temporal variation of carbon stocks in a lowland tropical forest in West Africa. For. Ecol. Manage. 289:10–17.

[ece32175-bib-0061] Loreau, M. , and A. Hector . 2001 Partitioning selection and complementarity in biodiversity experiments. Nature 412:72–76. 11452308 10.1038/35083573

[ece32175-bib-0062] Magnani, F. , M. Mencuccini , and J. Grace . 2000 Age‐related decline in stand productivity: the role of structural acclimation under hydraulic constraints. Plant, Cell Environ. 23:251–263.

[ece32175-bib-0063] Malhi, Y. , T. R. Baker , O. L. Phillips , S. Almeida , E. Alvarez , L. P. Arroyo , et al. 2004 The above‐ground coarse wood productivity of 104 Neotropical forest plots. Glob. Change Biol. 10:563–591.

[ece32175-bib-0064] Martin, A. R. , and S. C. Thomas . 2011 A reassessment of carbon content in tropical trees. PLoS ONE 6:e23533. 21858157 10.1371/journal.pone.0023533 PMC3157388

[ece32175-bib-0065] Martin, P. A. , J. Bullock , and A. Newton . 2013 Carbon pools recover more rapidly than plant biodiversity in secondary tropical forests. Proc. R. Soc. B 280:20132236. 10.1098/rspb.2013.2236 PMC3826225 24197410

[ece32175-bib-0066] Martin, P. A. , A. C. Newton , M. Pfeifer , M. Khoo , and J. M. Bullock . 2015 Impacts of tropical selective logging on carbon storage and tree species richness: a meta‐analysis. For. Ecol. Manage. 356:224–233.

[ece32175-bib-0067] Mercado, L. M. , S. Patino , T. F. Domingues , N. M. Fyllas , G. P. Weedon , S. Sitch , et al. 2011 Variations in Amazon forest productivity correlated with foliar nutrients and modelled rates of photosynthetic carbon supply. Philos. Trans. R. Soc. B 366:3316–3329. 10.1098/rstb.2011.0045 PMC3179632 22006971

[ece32175-bib-0068] Michaletz, S. T. , D. Cheng , A. J. Kerkhoff , and B. J. Enquist . 2014 Convergence of terrestrial plant production across global climate gradients. Nature 512:39–43. 25043056 10.1038/nature13470

[ece32175-bib-0069] Miller, S. D. , M. L. Goulden , L. R. Hutyra , M. Keller , S. R. Saleska , S. C. Wofsy , et al. 2011 Reduced impact logging minimally alters tropical rainforest carbon and energy exchange. Proc. Natl Acad. Sci. 108:19431–19435. 22087005 10.1073/pnas.1105068108 PMC3228459

[ece32175-bib-0070] Muller‐Landau, H. C. , M. Detto , R. A. Chisholm , S. P. Hubbell , and R. Condit . 2014 Detecting and projecting changes in forest biomass from plot data Pp. 381–415 in Coomes D. A., Burslem D. F. R. P. and Simonson W. D., eds. Forests and global change. Cambridge University Press, Cambridge.

[ece32175-bib-0071] Okuda, T. , M. Suzuki , N. Adachi , E. S. Quah , N. A. Husseion , and N. Manokaran . 2003 Effect of selective loggings on canopy and stand structure and tree species composition in a lowland dipterocarp forest in peninsular Malaysia. For. Ecol. Manage. 175:297–320.

[ece32175-bib-0072] Osazuwa‐Peters, O. L. , C. A. Chapman , and A. E. Zanne . 2015 Selective logging: does the imprint remain on tree structure and composition after 45 years? Conserv. Physiol. 3:1–12. 10.1093/conphys/cov012 PMC4778436 27293697

[ece32175-bib-0073] Pan, Y. , R. A. Birdsey , J. Fang , R. Houghton , P. E. Kauppi , W. A. Kurz , et al. 2011 A large and persistent carbon sink in the world's forests. Science 333:988–993. 21764754 10.1126/science.1201609

[ece32175-bib-0074] Paquette, A. , and C. Messier . 2011 The effect of biodiversity on tree productivity: from temperate to boreal forests. Glob. Ecol. Biogeogr. 20:170–180.

[ece32175-bib-0075] Poorter, L. , and F. Bongers . 2006 Leaf traits are good predictors of plant performance across 53 rain forest species. Ecology 87:1733–1743. 16922323 10.1890/0012-9658(2006)87[1733:ltagpo]2.0.co;2

[ece32175-bib-0076] Poorter, L. , M. T. van der Sande , J. Thompson , E. J. M. M. Arets , A. Alarcón , J. Álvarez‐Sánchez , et al. 2015 Diversity enhances carbon storage in tropical forests. Glob. Ecol. Biogeogr. 24:1314–1328.

[ece32175-bib-0077] Pretzsch, H. 2014 Canopy space filling and tree crown morphology in mixed‐species stands compared with monocultures. For. Ecol. Manage. 327:251–264.

[ece32175-bib-0078] Quesada, C. A. , O. L. Phillips , M. Schwarz , C. I. Czimczik , T. R. Baker , S. Patiño , et al. 2012 Basin‐wide variations in Amazon forest structure and function are mediated by both soils and climate. Biogeosciences 9:2203–2246.

[ece32175-bib-0079] R Core Development Team . 2013 R: a language and environment for statistical computing. R Foundation for Statistical Computing, Vienna, Austria.

[ece32175-bib-0080] Reich, P. B. 2012 Key canopy traits drive forest productivity. Proc. R. Soc. B 279:2128–2134. 10.1098/rspb.2011.2270 PMC3321697 22279168

[ece32175-bib-0081] Reich, P. B. 2014 The world‐wide “fast‐slow” plant economics spectrum: a traits manifesto. J. Ecol. 102:275–301.

[ece32175-bib-0082] Rosseel, Y. 2012 lavaan: an R package for structural equation modeling. J. Stat. Softw. 48:1–36.

[ece32175-bib-0083] Rüger, N. , and R. Condit . 2012 Testing metabolic theory with models of tree growth that include light competition. Funct. Ecol. 26:759–765.

[ece32175-bib-0084] Russo, S. E. , S. J. Davies , D. A. King , and S. Tan . 2005 Soil‐related performance variation and distributions of tree species in a Bornean rain forest. J. Ecol. 93:879–889.

[ece32175-bib-0085] Russo, S. E. , P. Brown , S. Tan , and S. J. Davies . 2008 Interspecific demographic trade‐offs and soil‐related habitat associations of tree species along resource gradients. J. Ecol. 96:192–203.

[ece32175-bib-0086] Ryan, M. G. , D. Binkley , and J. H. Fownes . 1997 Age‐related decline in forest productivity: pattern and process. Adv. Ecol. Res. 27:213–262.

[ece32175-bib-0087] Sapijanskas, J. , A. Paquette , C. Potvin , N. Kunert , and M. Loreau . 2014 Tropical tree diversity enhances light capture through crown plasticity and spatial and temporal niche differences. Ecology 95:2479–2492.

[ece32175-bib-0088] Schnitzer, S. A. , and F. Bongers . 2011 Increasing liana abundance and biomass in tropical forests: emerging patterns and putative mechanisms. Ecol. Lett. 14:397–406. 21314879 10.1111/j.1461-0248.2011.01590.x

[ece32175-bib-0089] Slik, J. W. F. , S. I. Aiba , F. Q. Brearley , C. H. Cannon , O. Forshed , K. Kitayama , et al. 2010 Environmental correlates of tree biomass, basal area, wood specific gravity and stem density gradients in Borneo's tropical forests. Glob. Ecol. Biogeogr. 19:50–60.

[ece32175-bib-0090] Slik, J. W. F. , G. Paoli , K. Mcguire , I. Amaral , J. Barroso , M. Bastian , et al. 2013 Large trees drive forest aboveground biomass variation in moist lowland forests across the tropics. Glob. Ecol. Biogeogr. 22:1261–1271.

[ece32175-bib-0091] Stephenson, N. L. , A. J. Das , R. Condit , S. E. Russo , P. J. Baker , N. G. Beckman , et al. 2014 Rate of tree carbon accumulation increases continuously with tree size. Nature 507:90–93. 24429523 10.1038/nature12914

[ece32175-bib-0092] Sterck, F. J. , L. Poorter , and F. Schieving . 2006 Leaf traits determine the growth‐survival trade‐off across rain forest tree species. Am. Nat. 167:758–765. 16671019 10.1086/503056

[ece32175-bib-0093] Sterck, F. , L. Markesteijn , F. Schieving , and L. Poorter . 2011 Functional traits determine trade‐offs and niches in a tropical forest community. Proc. Natl Acad. Sci. 108:20627–20632. 22106283 10.1073/pnas.1106950108 PMC3251078

[ece32175-bib-0094] Talbot, J. , S. L. Lewis , G. Lopez‐Gonzalez , R. J. W. Brienen , A. Monteagudo , T. R. Baker , et al. 2014 Methods to estimate aboveground wood productivity from long‐term forest inventory plots. For. Ecol. Manage. 320:30–38.

[ece32175-bib-0095] Tanner, E. V. J. , P. M. Vitousek , and E. Cuevas . 1998 Experimental investigation of nutrient limitation of forest growth on wet tropical mountains. Ecology 79:10–22.

[ece32175-bib-0096] Townsend, A. R. , C. C. Cleveland , G. P. Asner , and M. M. C. Bustamante . 2007 Controls over foliar N: P ratios in tropical rain forests. Ecology 88:107–118. 17489459 10.1890/0012-9658(2007)88[107:cofnri]2.0.co;2

[ece32175-bib-0097] Tsui, C.‐C. , Z.‐S. Chen , and C.‐F. Hsieh . 2004 Relationships between soil properties and slope position in a lowland rain forest of southern Taiwan. Geoderma 123:131–142.

[ece32175-bib-0098] Vilà, M. , A. Carrillo‐Gavilán , J. Vayreda , H. Bugmann , J. Fridman , W. Grodzki , et al. 2013 Disentangling biodiversity and climatic determinants of wood production. PLoS ONE 8:e53530. 23437038 10.1371/journal.pone.0053530 PMC3577818

[ece32175-bib-0099] Vitousek, P. M. , S. Porder , B. Z. Houlton , and O. A. Chadwick . 2010 Terrestrial phosphorus limitation: mechanisms, implications, and nitrogen‐phosphorus interactions. Ecol. Appl. 20:5–15. 20349827 10.1890/08-0127.1

[ece32175-bib-0100] West, T. A. P. , E. Vidal , and F. E. Putz . 2014 Forest biomass recovery after conventional and reduced‐impact logging in Amazonian Brazil. For. Ecol. Manage. 314:59–63.

[ece32175-bib-0101] Whitmore, T. C. 1990 An introduction to tropical rainforests. Clarendon Press, Oxford.

[ece32175-bib-0102] Zanne, A. E. , G. Lopez‐Gonzalez , D. A. Coomes , J. Ilic , S. Jansen , S. L. Lewis , et al. 2009 Global wood density database. Dryad Digital Repository. doi:10.5061/dryad.234.

[ece32175-bib-0103] Zhang, Y. , and H. Y. H. Chen . 2015 Individual size inequality links forest diversity and above‐ground biomass. J. Ecol. 103:1245–1252.

