# Supplementary material for: Drivers of aboveground wood production in a lowland tropical forest of West Africa: teasing apart the roles of tree density, tree diversity, soil phosphorus, and historical logging
Source: Ecol Evol. 2016 May 18;6(12):4004–17. doi: 10.1002/ece3.2175 (PMC4875916; doi:10.1002/ece3.2175)
Supplement: Supplementary file 1 — Appendix S1. Soil data. Appendix S2. Estimating AWP from permanent plot data. Appendix S3. Correlations among model predictors. [file ECE3-6-4004-s001.docx]

**Supporting information**

[Appendix S1 | Soil data 2](#_Toc447884927)

[Soil sampling 2](#_Toc447884928)

[Laboratory analysis 2](#_Toc447884929)

[Environmental and spatial variability in soil P 2](#_Toc447884930)

[Appendix S2 | Estimating AWP from permanent plot data 4](#_Toc447884931)

[Individual tree growth models 4](#_Toc447884932)

[Height – diameter allometry 6](#_Toc447884933)

[Comparing AWP estimates based on field data and model predictions 7](#_Toc447884934)

[Comparing SEM and multiple regression 9](#_Toc447884935)

[Appendix S3 | Correlations among model predictors 10](#_Toc447884936)

[References 11](#_Toc447884937)

## Appendix S1 | Soil data

### Soil sampling

Soil samples were collected from 48 of the 142 forest plots for the purpose of estimating soil carbon content and quantifying soil nutrient availability. In each plot, three soil samples were collected with the aid of a soil corer. After having removed litter from the sampling location, the soil corer was inserted to a standard depth of 20 cm. The three replicate soil samples collected were aggregated and placed into a sealable plastic bag marked with the unique identifier code of the plot. In addition to the soil cores, a 25 cm deep pit was dug in each sampled plot in order to collect samples for estimating soil bulk density. A metal ring of known volume (113.1 cm^3^) was placed at a depth of 5 cm and hammered into the side of the pit. The ring was then carefully extracted and the soil sample placed in a sealable plastic bag marked with the unique identifier code of the plot. This process was then repeated at a depth of 15 cm. Soil samples were allowed to air dry before being transported to the UK where soil analyses were performed.

### Laboratory analysis

Soil analyses were conducted in the Department of Geography of the University of Cambridge. For an overview of the protocols and equipment used in this laboratory see: <http://www.geog.cam.ac.uk/facilities/laboratories/techniques/>. Here we describe the approach used to quantify soil carbon (C), bulk density and soil phosphorus (P), although only soil P was considered in the analyses presented in the main text.

**Soil carbon (C)** **and** **bulk density**: Loss on ignition (LOI) was used to quantify soil C content and determining bulk density. Samples were first oven-dried at 105°C until a constant weight was achieved. To estimate bulk density, samples were first sifted through a 2 mm sieve, and the <2 mm fraction was then weighed with an electronic balance. Soil bulk density (in g cm^-3^) was then determined by dividing the weight of the <2 mm fraction by the volume of the ring used to collect the bulk density samples (113.1 cm^3^). To quantify soil C, oven-dried samples were placed in a muffle furnace and progressively heated to 400°C, 480°C, 550°C and 950°C to remove organic matter and break down carbonate. An electronic balance was used to determine the loss in mass at each stages of the analysis, allowing soil C content (%) to be determined.

**Soil phosphorus** **(P)**: A sample measuring 1 cm^3^ was digested using nitro-hydrochloric acid (*aqua regia*), then made up to 50 ml using polished water before being diluted 10 fold with polished water. Inductively coupled plasma optical emission spectrometry (ICP-OES) was then used to measure phosphates (total soil P; expressed in parts per million).

### Environmental and spatial variability in soil P

Due to logistical constraints, soil samples were not collected for all forest plots considered in our analysis (only for 48 of the 142 plots). In order to account for the effects of soil P on AWP across all plots, we therefore used the samples that were collected to infer soil P for plots where no soil data were available. For this purpose we developed a mixed-effects model in which soil P was expressed as function of physical and spatial plot-level attributes. Specifically, we modelled soil P in relation to terrain slope (estimated using a clinometer), elevation (obtained from GPS data) and distance from streams (estimated in a GIS environment), and allowed soil P baseline levels to vary among plots clustered within a transect (i.e., random intercept term). We then used the model to predict soil P for all plots where soils were not sampled. The model was able to adequately capture variation in soil P among plots (Fig. S1a; Pearson’s correlation between predicted and observed values = 0.62, *P* < 0.0001). In particular, we found that soil P increased closer to rivers and was greatest in low-lying plots with level terrain. Based on the distribution of measured soil P values and previous work on P-limitation in tropical forests (Quesada *et al.* 2012), we categorized plots into one of three soil P classes (Fig. S1b): low (<300 mg P kg^-1^; 36 plots), medium (300 – 500 mg P kg^-1^; 61 plots) and high soil P (>500 mg P kg^-1^; 45 plots). This classification was adopted in all further modelling, although it should be noted that quantitatively similar results were obtained when applying alternative thresholds (e.g., equal number of plots per soil P class).


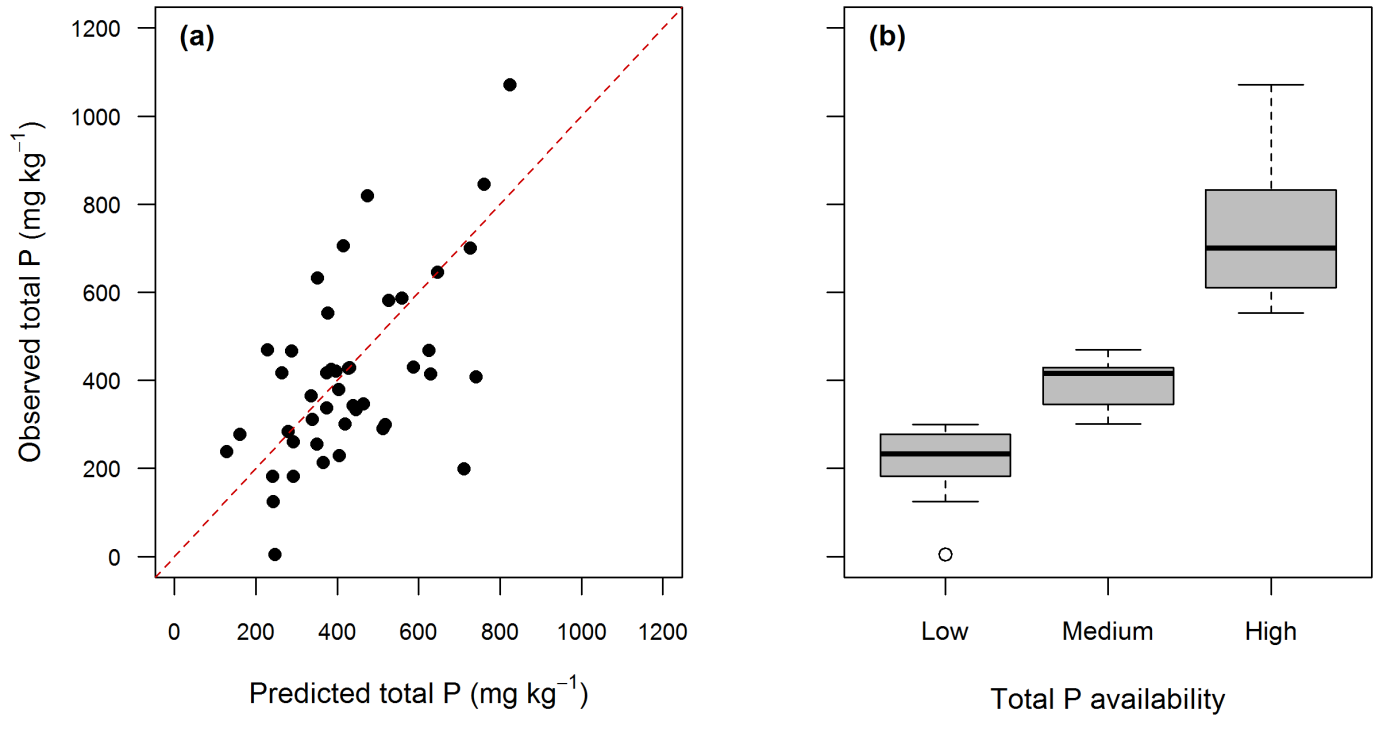


**Fig. S1** | Relationship between measured total P and (**a**) values predicted based on mixed-effects models and (**b**) boxplots showing how measured total P values vary among the three soil P classes.

## Appendix S2 | Estimating AWP from permanent plot data

### Individual tree growth models

We compared a number of alternative and increasing complex growth functions for the purpose of modelling the diameter growth of individual trees (Table S1). Three main factors were considered in modelling diameter growth: the relationship between tree growth and size (Fig. S2a), the effects of competition for light on growth (Fig. S2b), and variation in growth among trees belonging to different genera (grey lines in Fig. S2). Growth functions shown in Table S1 were parametrised using non-linear mixed effects models as implemented in the *nlme* R package, and were compared against each other on the basis of AIC. Of the models we tested, M6 was selected for the purpose of modelling tree diameter growth (Table S1). While M6 did not have a lower AIC than the comparatively simpler function of M5 (ΔAIC = 0.5), we chose the more complex of the two models as it allows for a more realistic response of tree growth to light availability (Coomes & Allen 2007; Coomes *et al.* 2012).


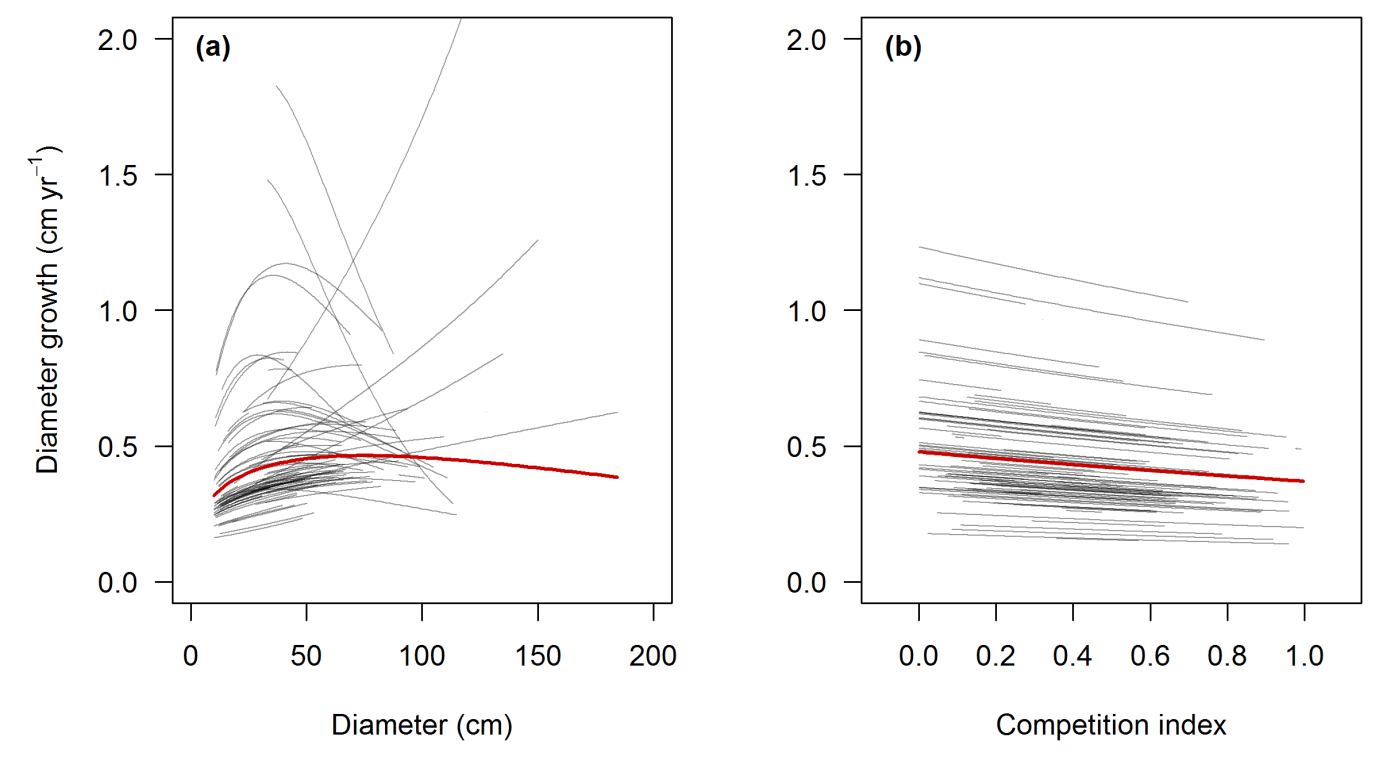


**Fig. S2** | Relationship between diameter growth and (**a**) tree diameter and (**b**) competition index (scaled between 0 – 1 to aid model convergence). Fitted curves show relationships as modelled by M6 in Table S1, and were obtained by allowing one variable to vary [e.g., diameter in the case of panel (**a**)] while the other is kept constant at its mean value (i.e., mean competition index across all trees). Red curves correspond to fitted relationships across taxonomic groups, while grey lines illustrate variation in the response among different genera of trees.

**Table S1** | Parameter estimates for alternative models of tree diameter growth. Diameter growth was expressed as a function of tree size (*D*) and competition with larger neighbours (*B_L_*). Furthermore, we tested whether growth curves varied among taxonomic groups by allowing parameter estimates to vary among genera in a mixed-effects modelling framework. Models were compared on the basis of AIC and the last column gives the AIC of each model relative to that of M0. ^†^Coomes & Allen (2007); ^‡^Coomes *et al.* (2012).

|  | Model component | | |  | Parameter estimates (± SE) | | | | |  |
| --- | --- | --- | --- | --- | --- | --- | --- | --- | --- | --- |
| Model | Size | Competition | Taxonomy | Growth function | *ρ_0_* | *ρ_1_* | *ρ_2_* | *ρ_3_* | *ρ_4_* | ΔAIC |
| M0 |  |  |  | ${G=\rho}_{0}$ | 0.43 (0.01) |  |  |  |  | 0 |
| M1 | ✓ |  |  | ${G=\rho}_{0}D^{\rho_{1}}$ | 0.09 (0.02) | 0.45 (0.06) |  |  |  | –52.5 |
| M2 | ✓ |  | ✓ | ${G=\rho}_{0}D^{\rho_{1}}$ | 0.17 (0.04) | 0.26 (0.07) |  |  |  | –175.9 |
| M3 | ✓ |  | ✓ | ${G=\rho}_{0}D^{\rho_{1}}\exp(\rho_{2}D)$ | 0.17 (0.08) | 0.27 (0.07) | 0.002 (0.005) |  |  | –192.5 |
| M4 | ✓ | ✓ | ✓ | $G=\frac{\rho_{0}D^{\rho_{1}}}{1+\rho_{3}B_{L}}$ | 0.26 (0.08) | 0.18 (0.07) |  | 0.38 (0.20) |  | –177.2 |
| M5 | ✓ | ✓ | ✓ | $G=\frac{\rho_{0}D^{\rho_{1}}\exp(\rho_{2}D)}{1+\rho_{3}B_{L}}$ | 0.20 (0.10) | 0.31 (0.19) | –0.003 (0.005) | 0.63 (0.23) |  | –198.6 |
| M6 | ✓ | ✓ | ✓ | $G=\frac{\rho_{0}D^{\rho_{1}}\exp(\rho_{2}D)}{1+\rho_{3} \exp(\rho_{4}B_{L})}$ | 0.13 (0.07) | 0.33 (0.19) | –0.004 (0.005) | –0.37 (0.14) | –2.23 (2.62) | –198.1 |

### Height – diameter allometry

Instead of adopting a published height – diameter (*H–D*) allometry for the purpose of estimating tree heights (e.g., Feldpausch *et al.* 2012), we used *H* and *D* measurements made for 336 trees in Gola to parametrize our own *H–D* function (Small 1953; Lindsell & Klop 2013). A Weibull function was used to express the relationship between *H* and *D* (Feldpausch *et al.* 2012), as this functional form has been shown to better approximate *H* for large trees (e.g., compared to a power-law function; Fig. S3):

| $H=\rho_{5}\left( 1-\exp\left( {-\rho}_{6}D^{\rho_{7}} \right) \right)$ |  | (S1) |
| --- | --- | --- |

where *ρ_5–7_* are parameters to be estimated. The *H–D* model was fit using the *nls* function in R. Parameter estimates (± SE) were as follows: *ρ_5_* = 79.9 (25.2); *ρ_6_* = 0.011 (0.002); *ρ_7_* = 0.74 (0.06). The *H–D* equation we derived showed a considerably better fit to the data compared to the *H–D* equation for West African forests available in Feldpausch *et al.* (2012) (Fig. S3).


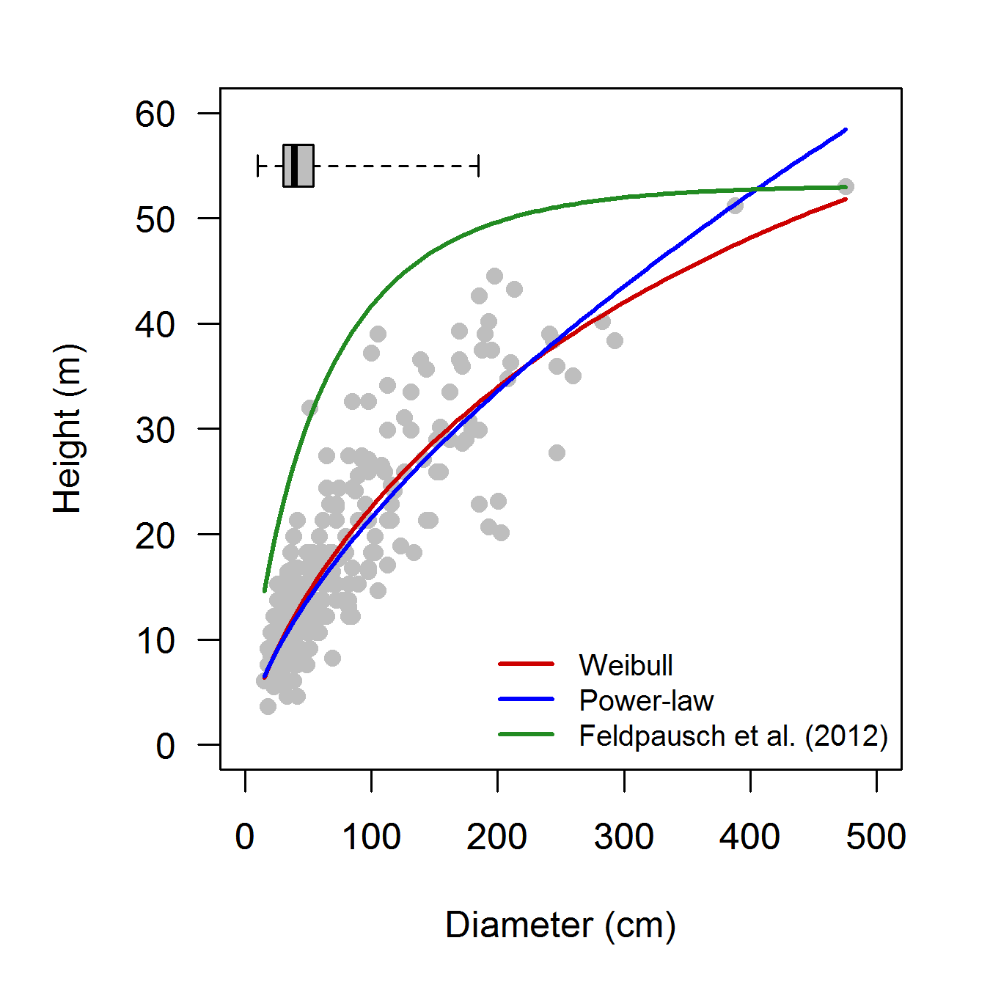


**Fig. S3** | Relationship between tree height and diameter for 336 trees from the Gola Rainforest National Park as reported in Small (1953). The fit of a Weibull function (red line) is compared to that of a power-law function (blue line) and to the fit of Feldpausch *et al.* (2012) height (H) – diameter (D) equation for West African forests (green line), where $H=53.133\left( 1-\exp\left( -0.0331D^{0.8329} \right) \right)$. The boxplot in the top left-hand corner shows the range and distribution of stem diameters of trees surveyed in this study.

### Comparing AWP estimates based on field data and model predictions

In addition to estimating AWP using the statistical modelling approach described in the main text, we also derived estimates of AWP by relying directly on field measurements. For this purpose we followed the protocol proposed by Talbot *et al.* (2014). Trees for which annual diameter increments exceeded 4 cm yr^-1^ or whose diameter decreased by more than 0.5 cm between censuses were assumed to have been incorrectly measured at one or more census periods. For these trees raw diameter increments were replaced with average diameter increment values of trees belonging to the same size class, with size class defined as 10 ≤ *D* < 20 cm, 20 ≤ *D* < 40 cm and *D* ≥ 40 cm. This same approach was used to estimate the growth of trees that died between censuses and of those that recruited. Estimates of AWP obtained in this way closely matched those reported in the main text (Fig S4). Furthermore, when we replaced modelled AWP estimates with field-based ones in the structural equation models (SEM), we obtained quantitatively similar results to those reported in the main text (Fig. S5). Basal area remained the strongest single driver of AWP, with both soil phosphorus and tree species diversity emerging as positive drivers of AWP (although in this instance the direct effect of tree species diversity on AWP was only marginally significant; *P* = 0.09).


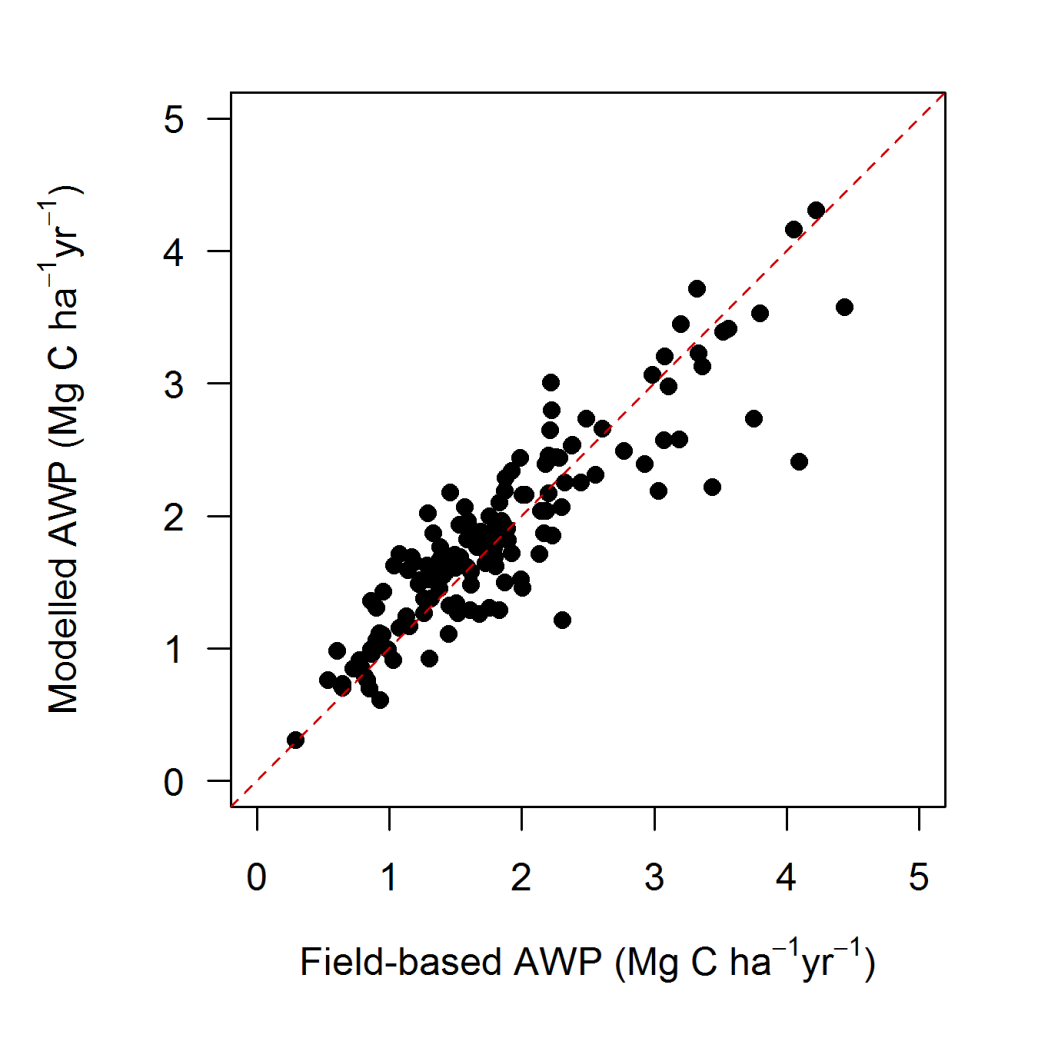


**Fig. S4** | Relationship between AWP estimated directly from field measurements and AWP estimates obtained from the individual tree growth models. The two approaches yield very similar estimates of AWP (Pearson’s correlation coefficient = 0.92).


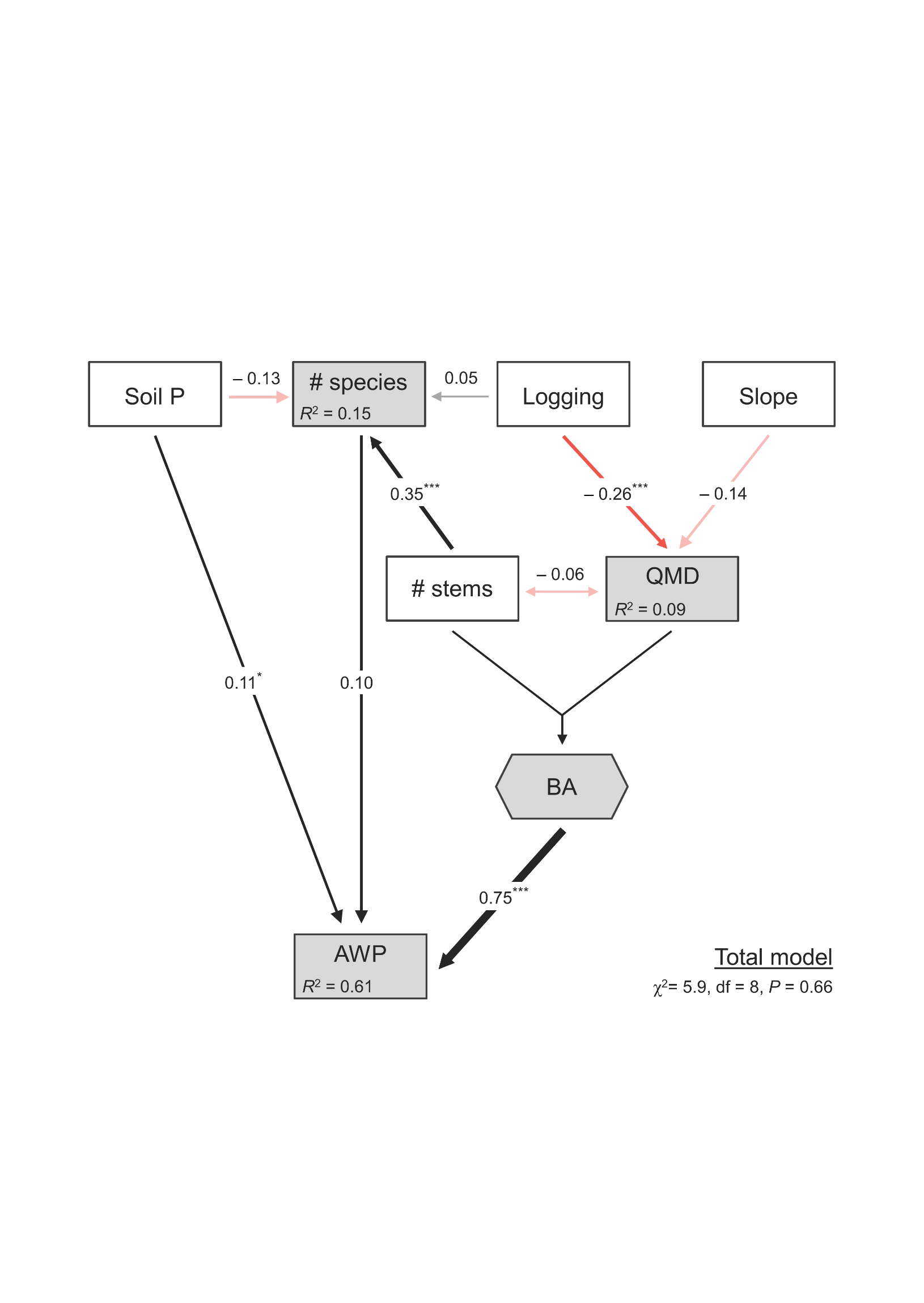


**Fig. S5** | Structural equation model relating variation in aboveground wood production (AWP) as estimated directly from field data to basal area (BA), effective number of tree species (# species) and soil phosphorus (P). BA was modelled as a composite variable of quadratic mean diameter (QMD) and stem density (# stems), which together determine BA exactly [see equation (4) in the main text]. Tree diversity is expressed as a function of stem density, soil P and past logging, which in conjunction with terrain slope is also assumed to influence QMD. Exogenous variables are represented by white boxes, while endogenous variables are shaded in grey. The width of the arrows reflects the strength of the pathway and is proportional to the standardized path coefficient (which is reported for each pathway). Black arrows denote positive relationships, while red arrows correspond to negative ones. Note that a bidirectional arrow is used to report the estimated co-variance between stem density and QMD. Asterisks denote significance levels of the pathways in the model (* *P* < 0.05; ** *P* < 0.01; *** *P* < 0.001). *R^2^* values are reported for each endogenous variable and model fit statistics are given in the bottom right-hand corner.

### Comparing SEM and multiple regression

To evaluate the robustness of the results obtained from the structural equation modelling (SEM) approach presented in the main text, we used multiple regression as a complementary analytical tool to examine what drives variation in aboveground wood production (AWP) among plots. Specifically we modelled AWP (log transformed) as a function of plot basal area (BA; also log transformed), effective number of species, past logging (binary variable; yes/no), terrain slope and soil phosphorus (P) content (discrete variable with three levels; low, medium and high). The results of the multiple regression model were strongly consistent with those of the SEM. Overall, the multiple regression model explained 80% of the variation in AWP among plots. Of the five explanatory variables included in the model, the strongest determinant of AWP was BA (slope = 0.76 ± 0.07; *P* < 0.0001). Both tree diversity (slope = 0.029 ± 0.017; *P* = 0.0015) and soil P content (slope = 0.055 ± 0.040; *P* = 0.0073) also emerged as significantly positive drivers of AWP. By contrast, both terrain slope and past logging did not directly influence AWP rates across Gola forest.

## Appendix S3 | Correlations among model predictors


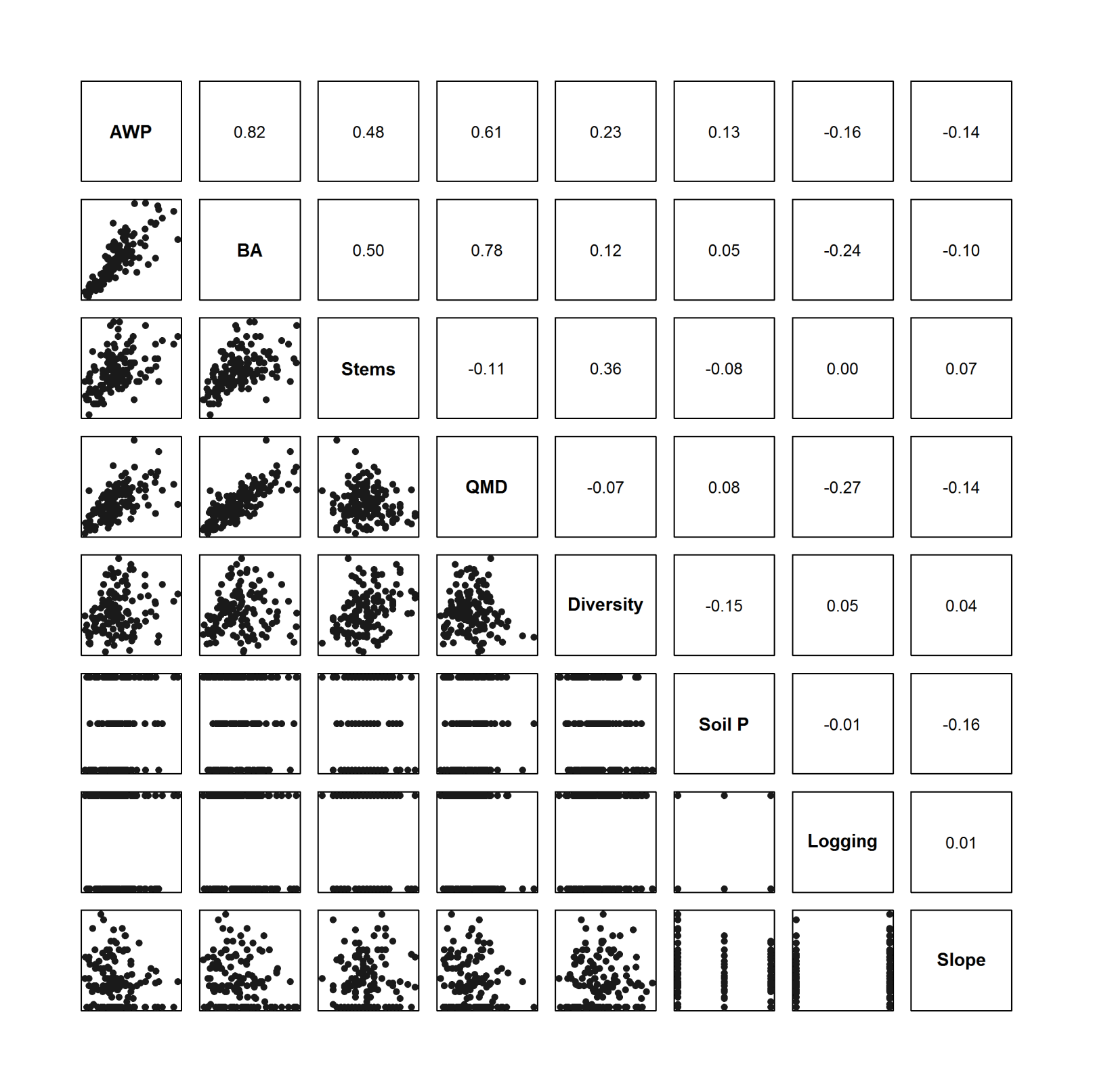


**Fig. S6** | Correlation plots between AWP and the predictors used in structural equation models. Pearson’s correlation coefficients are printed for each pair-wise comparison.

## References

Coomes, D.A. & Allen, R.B. (2007) Effects of size, competition and altitude on tree growth. *Journal of Ecology*, **95**, 1084–1097.

Coomes, D.A., Holdaway, R.J., Kobe, R.K., Lines, E.R. & Allen, R.B. (2012) A general integrative framework for modelling woody biomass production and carbon sequestration rates in forests. *Journal of Ecology*, **100**, 42–64.

Feldpausch, T.R., Lloyd, J., Lewis, S.L., Brienen, R.J.W., Gloor, M., Monteagudo Mendoza, A., Lopez-Gonzalez, G., Banin, L., Abu Salim, K., Affum-Baffoe, K., Alexiades, M., Almeida, S., Amaral, I., Andrade, A., Aragão, L.E.O.C., Araujo Murakami, A., Arets, E.J.M., Arroyo, L., Aymard C., G.A., Baker, T.R., Bánki, O.S., Berry, N.J., Cardozo, N., Chave, J., Comiskey, J.A., Alvarez, E., De Oliveira, A., Di Fiore, A., Djagbletey, G., Domingues, T.F., Erwin, T.L., Fearnside, P.M., França, M.B., Freitas, M.A., Higuchi, N., Honorio C., E., Iida, Y., Jiménez, E., Kassim, A.R., Killeen, T.J., Laurance, W.F., Lovett, J.C., Malhi, Y., Marimon, B.S., Marimon-Junior, B.H., Lenza, E., Marshall, A.R., Mendoza, C., Metcalfe, D.J., Mitchard, E.T.A., Neill, D. a., Nelson, B.W., Nilus, R., Nogueira, E.M., Parada, A., S.-H. Peh, K., Pena Cruz, A., Peñuela, M.C., Pitman, N.C.A., Prieto, A., Quesada, C.A., Ramírez, F., Ramírez-Angulo, H., Reitsma, J.M., Rudas, A., Saiz, G., Salomão, R.P., Schwarz, M., Silva, N., Silva-Espejo, J.E., Silveira, M., Sonké, B., Stropp, J., Taedoumg, H.E., Tan, S., Ter Steege, H., Terborgh, J., Torello-Raventos, M., Van Der Heijden, G.M.F., Vásquez, R., Vilanova, E., Vos, V.A., White, L., Willcock, S., Woell, H. & Phillips, O.L. (2012) Tree height integrated into pantropical forest biomass estimates. *Biogeosciences*, **9**, 3381–3403.

Lindsell, J.A. & Klop, E. (2013) Spatial and temporal variation of carbon stocks in a lowland tropical forest in West Africa. *Forest Ecology and Management*, **289**, 10–17.

Quesada, C.A., Phillips, O.L., Schwarz, M., Czimczik, C.I., Baker, T.R., Patiño, S., Fyllas, N.M., Hodnett, M.G., Herrera, R., Almeida, S., Alvarez Dávila, E., Arneth, A., Arroyo, L., Chao, K.J., Dezzeo, N., Erwin, T., Di Fiore, A., Higuchi, N., Honorio Coronado, E., Jimenez, E.M., Killeen, T., Lezama, A.T., Lloyd, G., Löpez-González, G., Luizão, F.J., Malhi, Y., Monteagudo, A., Neill, D.A., Núñez Vargas, P., Paiva, R., Peacock, J., Peñuela, M.C., Peña Cruz, A., Pitman, N., Priante Filho, N., Prieto, A., Ramírez, H., Rudas, A., Salomão, R., Santos, A.J.B., Schmerler, J., Silva, N., Silveira, M., Vásquez, R., Vieira, I., Terborgh, J. & Lloyd, J. (2012) Basin-wide variations in Amazon forest structure and function are mediated by both soils and climate. *Biogeosciences*, **9**, 2203–2246.

Small, D. (1953) *Some Ecological and Vegetational Studies in the Gola Forest Reserve, Sierra Leone, B.W. Africa*. Queen’s University, Belfast.

Talbot, J., Lewis, S.L., Lopez-Gonzalez, G., Brienen, R.J.W., Monteagudo, A., Baker, T.R., Feldpausch, T.R., Malhi, Y., Vanderwel, M., Araujo-Murakami, A., Arroyo, L.P., Chao, K.J., Erwin, T., van der Heijden, G., Keeling, H., Killeen, T., Neill, D., Núñez Vargas, P., Parada Gutierrez, G.A., Pitman, N., Quesada, C.A., Silveira, M., Stropp, J. & Phillips, O.L. (2014) Methods to estimate aboveground wood productivity from long-term forest inventory plots. *Forest Ecology and Management*, **320**, 30–38.
